# GNN4DM: a graph neural network-based method to identify overlapping functional disease modules

**DOI:** 10.1093/bioinformatics/btae573

**Published:** 2024-09-25

**Authors:** András Gézsi, Péter Antal

**Affiliations:** Department of Artificial Intelligence and Systems Engineering, Budapest University of Technology and Economics, Budapest H-1117, Hungary; Department of Artificial Intelligence and Systems Engineering, Budapest University of Technology and Economics, Budapest H-1117, Hungary

## Abstract

**Motivation:**

Identifying disease modules within molecular interaction networks is an essential exploratory step in computational biology, offering insights into disease mechanisms and potential therapeutic targets. Traditional methods often struggle with the inherent complexity and overlapping nature of biological networks, and they are limited in effectively leveraging the vast amount of available genomic data and biological knowledge. This limitation underscores the need for more effective, automated approaches to integrate these rich data sources.

**Results:**

In this work, we propose GNN4DM, a novel graph neural network-based structured model that automates the discovery of overlapping functional disease modules. GNN4DM effectively integrates network topology with genomic data to learn the representations of the genes corresponding to functional modules and align these with known biological pathways for enhanced interpretability. Following the DREAM benchmark evaluation setting and extending with three independent data sources (GWAS Atlas, FinnGen, and DisGeNET), we show that GNN4DM performs better than several state-of-the-art methods in detecting biologically meaningful modules. Moreover, we demonstrate the method’s applicability by discovering two novel multimorbidity modules significantly enriched across a diverse range of seemingly unrelated diseases.

**Availability and implementation:**

Source code, all training data, and all identified disease modules are freely available for download at https://github.com/gezsi/gnn4dm. GNN4DM was implemented in Python.

## 1 Introduction

It’s widely recognized that molecular networks exhibit a high degree of modularity, meaning, there exist subgroups of nodes that are more densely interconnected than would be anticipated by random chance. Often, these individual modules encapsulate genes or proteins that partake in identical or closely related biological functions (Hartwell *et al.* 1999, Mitra *et al.* 2013). This characteristic has been widely leveraged to infer unknown gene functions, a principle termed “guilt-by-association” (Sharan *et al.* 2007). Similarly, genes associated with the same disease or symptom tend to cluster together in molecular networks, as demonstrated in various studies (Oti and Brunner 2007, Menche *et al.* 2015, Chagoyen and Pazos 2016).

By convention, a *disease module* is defined as a topological network module associated with a disease, specifically a network module whose malfunctioning components (genes or proteins) contribute to the abnormal phenotype associated with that disease (Loscalzo and Barabasi 2011). Identifying disease modules within molecular interaction networks has contributed to uncovering the genetic basis of a wide range of complex diseases, offering insights into their underlying molecular mechanisms (Manners *et al.* 2018, Wang *et al.* 2020), facilitating the identification of potential therapeutic targets (Zhao and Li 2012), and in various other tasks, such as quantifying the associations between diseases (Ni *et al.* 2020).

Traditionally, disease module identification aims to uncover cohesive modules (also known as communities or clusters) with denser internal connections compared to external ones. However, identifying disease modules is an inherently ill-posed problem, as the interactome, similarly to most real networks, lacks a clear community structure, yet it is characterized by well-defined statistics of overlapping and nested communities (Palla *et al.* 2005).

In this study, we adopt a more integrative approach toward modules, targeting the identification of *functional disease modules* that are not merely cohesive but also collectively contribute to higher-level biological functions reflecting the modular and hierarchical organization observed in biological systems. Our proposed method hinges on two core modeling assumptions:

Assumption 1. *Functional disease modules are overlapping.* Functional modules in biological networks often exhibit a significant degree of overlap. This overlapping nature is indicative of the multi-functionality of genes, where a single gene can be part of multiple functional groups or pathways. This assumption is well-acknowledged, with many studies reporting an overlapping community structure across various biological networks (Gavin *et al.* 2002, Loscalzo and Barabasi 2011).

Assumption 2. *Functional modules are organized into a complex, hierarchical system that encompasses known biological pathways.* We posit that either (i) biological pathways are orchestrated by smaller, cohesive functional modules, or (ii) these pathways themselves interact, potentially serving as components of even larger functional modules. In either case, the functional modules represent clusters of genes working together to execute specific biological functions. This modular organization is not only a hallmark of biological complexity but also a manifestation of functional redundancy and robustness in biological systems (Ravasz *et al.* 2002, Soyer 2007, Clune *et al.* 2013, Mengistu *et al.* 2016, Hatleberg and Hinman 2021).

Disease module identification approaches are broadly categorized into general and disease-specific methods. General methods aim to reveal disease modules based on the topology of molecular networks without tailoring them to any particular disease [see Manipur *et al.* (2023) for a recent review]. In contrast, disease-specific methods aim to uncover disease-pertinent modules using disease-specific data, like genome-wide association signals or cancer mutation data. Our proposed approach aligns with general methods.

This study primarily focuses on methods based on graph neural networks (GNNs). GNNs have emerged as effective tools for leveraging graph-structured data. They efficiently capture the relationships among nodes within a network while also incorporating node attributes, achieving state-of-the-art results in various tasks. NOCD, introduced by Shchur and Günnemann (2019), was one of the pioneering neural models for overlapping community detection. It combines the power of GNNs with the Bernoulli–Poisson graph generative probabilistic model. This model learns a nonnegative community affiliation matrix through a GNN, which represents the nodes’ assignment into communities. Deepgmd uses a similar formalism to NOCD aiming to detect gene regulatory modules within gene co-expression networks derived from gene expression profile data (Ye *et al.* 2022). UCoDe, introduced by Moradan *et al.* (2023), performs both overlapping and nonoverlapping community detection. It utilizes a contrastive loss function that maximizes a soft version of network modularity, offering a unified approach to community detection in networks.

Another line of research focuses on deriving patient-specific disease subnetworks to capture individualized disease mechanisms using GNNs. Methods like GNN-SubNet (Pfeifer *et al.* 2022, 2023) and CGMega (Li *et al.* 2024) address this by integrating multi-omics patient-specific data to identify disease-associated subnetworks. However, because these methods rely on patient-specific data, they fundamentally differ from our approach, designed to identify *general* overlapping functional disease modules within a biological network. For further related methods, see e.g. Jia *et al.* (2019), Zhang *et al.* (2020), Chen *et al.* (2022), Yuan *et al.* (2023).

In this work, we propose a deep learning-based method, termed Graph Neural Network for Disease Modules (GNN4DM), that leverages these assumptions to identify overlapping functional disease modules that align well with known biological pathways. By using a two-step approach, initially, a Graph Convolutional Network (GCN) is utilized to fuse network structure and genomic data (comprising gene expression and genome-wide association data) to generate a community affiliation matrix representing the likelihood of each gene belonging to various modules. Subsequently, the identified modules are refined by leveraging known pathway information from various pathway databases utilizing constrained logistic regression models, aiming to align the modular structure with established biological knowledge. In short, the learned latent representations of the genes are influenced by two simultaneous information sources within a unified model: Genomic data through the network structure at the input and known pathway data through the mixture loss at the output. The two-component model is trained end-to-end to derive interpretable functional disease modules.

## 2 Materials and methods

### 2.1 Datasets

The architectural design of our model is based on using two different types of datasets. Primarily, datasets encompassing genome-wide information, along with structural properties of the nodes in the underlying graph, were utilized as input features in the model. Conversely, annotations from pathway databases possessing partial, specialized, and curated information were leveraged as auxiliary tasks for the model to predict.

#### 2.1.1 Data source for graph structure: protein–protein interactions

We utilized protein–protein interaction (PPI) data from STRING-DB v12.0 (Szklarczyk *et al.* 2019), following a comprehensive study by Huang *et al.* (2018) that assessed 21 human tissue-unaware interaction networks for their capability in predicting disease genes, and identified STRING as one of the top-performing networks. We mapped the protein identifiers to their corresponding Ensembl gene identifiers using the Ensembl Biomart tool. We selected only the highly confident interactions by filtering them based on their combined score, keeping those above 0.7. The resulting pairwise interactions defined the structure of the unweighted graph in which we identified the modules. The graph nodes represented 15 793 protein-coding genes, and the edges denoted 234 179 functional interactions between these genes.

#### 2.1.2 Data sources used for constructing input features

**Gene expression measurement data.** We used genome-wide gene expression measurement data from the Genotype-Tissue Expression (GTEx) project (Aguet *et al.* 2020). We used the bulk RNA-seq tissue expression dataset from the GTEx Analysis V8, which contains median gene-level transcript per million (TPM) values across 54 human tissues. We log-transformed the raw TPM values and imputed the missing expression values for 218 genes absent in the GTEx dataset by the tissue-wise mean TPM values. We used the transformed expression values as part of the input features for the gene nodes.

**Gene-level genome-wide association data.** We utilized gene-level genome-wide association data from the GWAS Atlas project Release 3 (Watanabe *et al.* 2019), which encompasses 4756 publicly available GWAS summary statistics. We downloaded the dataset containing the gene-level *P*-values computed by the MAGMA software for 19 436 protein-coding genes. We split the dataset into two parts; 3545 summary statistics exclusive to studies other than UK Biobank were used as inputs, while the 1211 UK Biobank-specific summary statistics were utilized for evaluation purposes (see Section 2.4). We imputed the missing *P*-values for 519 genes absent in the dataset with 0.5. We computed the negative log-transformed *P*-values (the higher, the more significant) and performed a PCA transformation for dimensionality- and noise reduction of the resulting significance values. We used the first 512 transformed dimensions and used these as part of the input features for the gene nodes. The total variance explained by the 512 principal components was 0.718. Supplementary Fig. S1 shows the cumulative explained variance for up to 2048 principal components.

**Centrality measures of nodes within the network.** Additionally, we calculated five other metrics using the NetworkX Python package (Hagberg *et al.* 2008) to quantify the centrality or importance of nodes within the network. These consist of degree, betweenness, eigenvector, and closeness centrality, and the PageRank score.

All the above vectors were concatenated and standardized to form 571D input feature vectors for the gene nodes.

#### 2.1.3 Pathway databases

We derived gene sets from the MSigDB v2023.1 pathway datasets (Subramanian *et al.* 2005), namely the BioCarta, KEGG, Reactome, and WikiPathways databases encompassing canonical representations of biological processes compiled by domain experts. We filtered all datasets to those pathways containing at least 10 but no more than 500 genes in the graph. This resulted in 221 pathways for BioCarta (comprising 1324 unique genes), 186 pathways for KEGG (4889 genes), 1288 for Reactome (9905 genes), and 621 pathways for WikiPathways (7166 genes). The pathways in these datasets served as auxiliary tasks that the model was trained to predict.

We note that the STRING database includes a “database” channel incorporating well-established knowledge about protein complexes, pathways, and other functional connections from sources such as KEGG and Reactome, thereby introducing some redundancy in the learning process. However, we use the pathway databases described in this section for fine-tuning the modules and preventing overfitting, not for evaluating the model. Consequently, this redundancy does not introduce bias into the evaluations.

### 2.2 The GNN4DM framework

**Figure 1.**
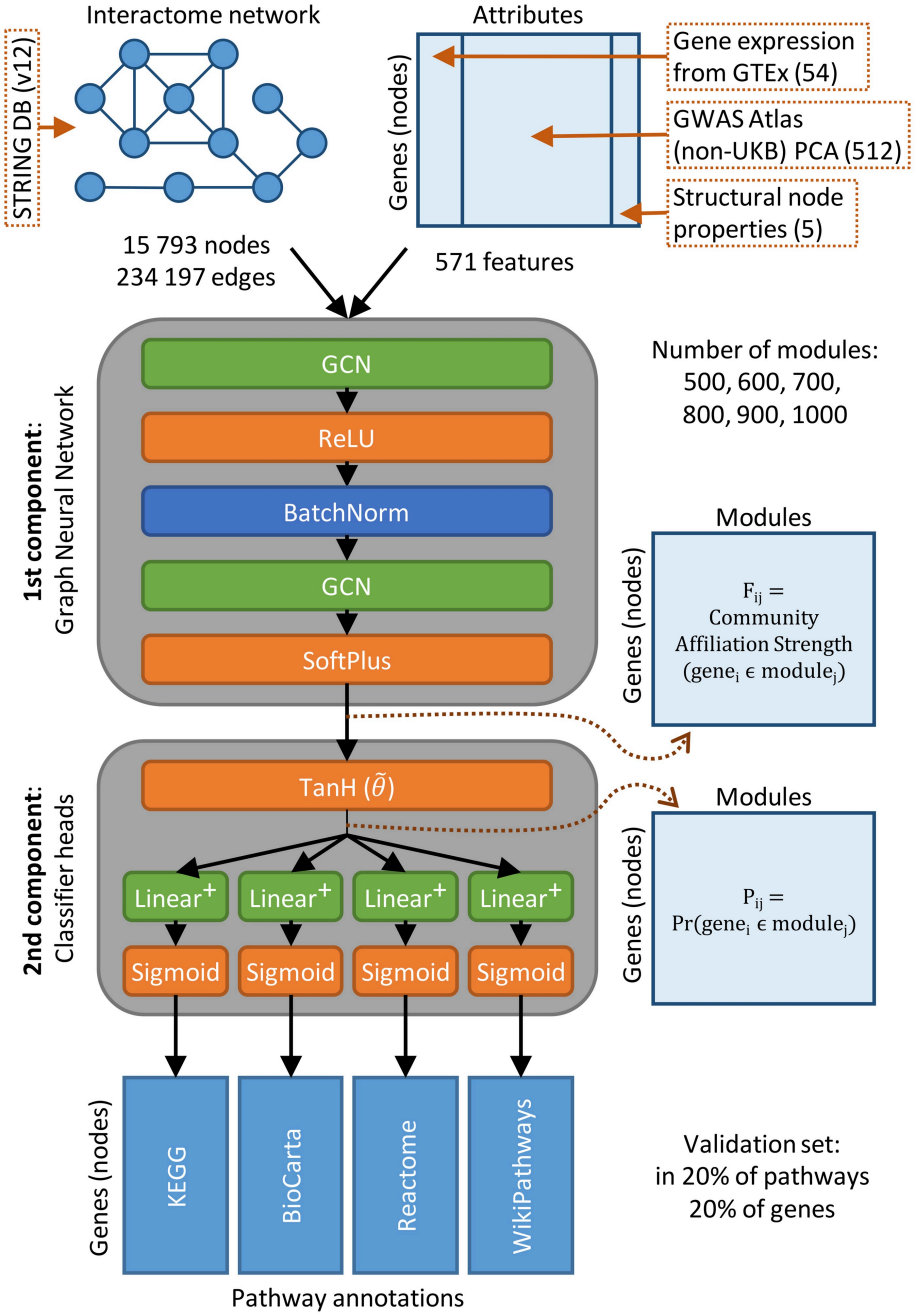
The GNN4DM framework. Datasets used for constructing the human interactome network (graph) and the attributes of the gene nodes in the graph are shown on top. The model is structured into two components. The first component of the framework uses a graph neural network to process the underlying graph and the genes’ input features to derive an inner representation for the nodes corresponding to their non-negative community affiliation strengths. The second component of the framework refines the inner representations (converted first into module membership probabilities) of the genes such that the identified modules align with biological pathways contained in various pathway databases. This component uses a constrained multivariable logistic regression to perform binary classification over each pathway in each pathway database. GCN: Graph Convolutional Network layer.

We created an interpretable, graph neural network-based framework called GNN4DM that is used to identify overlapping functional disease modules in a PPI network. The overview of the method is shown in Fig. 1.

**Notations.** Assuming an undirected, unweighted, static graph G(V,E) consisting of nodes *V* corresponding to human protein-coding genes and edges *E* representing the functional interactions between the corresponding gene nodes, we denote the binary adjacency matrix of *G* as $A\in {\left\{0,1\right\}}^{N\times N}$, where *N* is the number of nodes V={1,…,N} (*N* = 15 793), and $E=\{(u,v)\in V\times V:A_{uv}=1\}$ is the set of edges in the graph. Every node is associated with a *D*-dimensional input vector (*D* = 571) that is represented as a feature matrix $X\in R^{N\times D}$ for all nodes.

#### 2.2.1 Identifying overlapping modules via GNN layers

The main aim of the GNN4DM framework is to find overlapping modules in the graph. This is achieved by assigning the gene nodes into *M* number of modules where the assignment is represented by a nonnegative *community affiliation matrix* $F\in R_{\geq 0}^{N\times M}$, where $F_{um}$ represents the *strength* by which node *u* belongs to module *m*.

The first component of the GNN4DM framework uses Graph Convolutional Network layers (Kipf and Welling 2017) to generate the **F** community affiliation matrix, namely:

(1) $$F:={\text{GCN}}_{\theta }(A,X),$$

where θ represents the parameters of these layers. In our model, we used two GCN layers, applying a ReLU activation function and a batch normalization after the first layer, and a Softplus activation function after the second layer, which produces the nonnegative **F** affiliation weights.

Our method is based on the Bernoulli-Poisson graph generative model (Psorakis *et al.* 2011, Yang and Leskovec 2013, Shchur and Günnemann 2019), in which the entries of the adjacency matrix $A_{uv}$ are sampled according to a Bernoulli distribution parameterized by the community affiliations:

(2) $$A_{uv}∼\text{Bernoulli}(1-\text{exp}(-f_{u}f_{v}^{⊤})),$$

where $f_{u}=F_{u:}$ and $f_{v}=F_{v:}$ are the row vectors of the community affiliation matrix **F** of nodes *u* and *v*, respectively. Further details behind the rationale of this model can be found in the Supplementary Material.

We approximate the negative log-likelihood of the Bernoulli-Poisson model by the following loss:

(3) $$\begin{array}{cc} L_{BP}:=-\log(A|F)\approx -E_{(u,v)∼P_{E}}\left[\log(1-\text{exp}(-f_{u}f_{v}^{⊤})\right]\\ +E_{(u,v)∼P_{N}}\left[f_{u}f_{v}^{⊤}\right], & \end{array}$$

where $P_{E}$ and $P_{N}$ represent uniform distributions over edges and nonedges in the graph, respectively. These distributions are obtained through structured negative sampling. Specifically, for every positive edge sampled, a corresponding negative edge is also sampled, ensuring that the starting node is the same for both the positive and negative edges.

#### 2.2.2 Refining latent representations using auxiliary pathway information

The second component of the framework refines the gene representations, ensuring that the identified modules align to a well-calibrated degree (controlled by a hyper-parameter) with known biological pathways. Specifically, while the first component of the model processes genomic and network structure data to learn initial gene representations, the pathways from various databases are used as auxiliary tasks to fine-tune these representations. This is achieved through a multi-task learning approach, where the model simultaneously predicts pathway membership alongside identifying disease modules. This dual learning process enhances the alignment of the identified modules with known biological functions, thereby improving their biological relevance and interpretability.

The second component begins by converting the nonnegative module affiliations into probabilities, denoted as $\hat{P}$, which represent the probability of a gene belonging to a module. This conversion is achieved by applying a scaled hyperbolic tangent function element-wise to the community affiliation matrix **F**:

(4) $$\forall u,m:{\hat{P}}_{um}:={\text{tanh}}_{\tilde{\theta }}(F_{um})=\frac{\;\text{exp}(2\tilde{\theta }F_{um})+1}{\;\text{exp}(2\tilde{\theta }F_{um})-1},$$

where $\tilde{\theta }$ is a shared learnable scaling parameter.

Following this, for each *k*th pathway database (k∈{1,…,K}, where the *k*th pathway database contains $S^{\left(k\right)}$ number of pathways), individual linear layers are applied in parallel to the $\hat{P}$ module probabilities, each using a sigmoid activation function:

(5) $$\forall k:{\left[{\hat{Y}}^{\left(k\right)}\right]}^{T}:=\sigma \left(W^{\left(k\right)}{}^{T}{\hat{P}}^{T}+b^{\left(k\right)}\right),$$

where ${\hat{Y}}^{\left(k\right)}$ are the $N\times S^{\left(k\right)}$ dimensional predicted outputs of the linear layers, and $W^{\left(k\right)}$ and $b^{\left(k\right)}$ are the *k*th parallel linear layer’s weight matrix and bias vector, respectively. This step basically performs a binary classification over each individual pathway in each pathway database (i.e. a multi-label classification for each pathway database), where we denote the ground truth annotations as $Y^{\left(k\right)}\in {\left\{0,1\right\}}^{N\times S^{\left(k\right)}}$ in the *k*th pathway database, i.e. $Y_{ui}^{\left(k\right)}$ equals 1 if gene *u* is annotated with pathway *i* in the *k*th pathway database and 0 otherwise.

We use a binary cross entropy loss to measure the difference between the predictions and the ground truth, and compute their sum for all *K* pathway databases and for each pathway, i.e.

(6) $$L_{\mathrm{BCE}}=\sum_{k=1}^{K}\sum_{i=1}^{S^{\left(k\right)}}\text{BCE}\left({\hat{Y}}_{:i}^{\left(k\right)},Y_{:i}^{\left(k\right)}\right)$$

Note that the weights of the final parallel linear layers are constrained to be nonnegative, denoted as $W^{\left(k\right)}\in R_{\geq 0}^{M\times S^{\left(k\right)}}$, whereas the $b^{\left(k\right)}\in R^{S^{\left(k\right)}}$ biases of these layers are not subject to this constraint. Essentially, these final parallel layers function as *multivariable logistic regressions*, providing a coherent interpretation. Namely, the weight $W_{mi}^{\left(k\right)}$ quantifies the increase in log odds of a gene being part of the *i*th pathway in the *k*th pathway database, given that the gene belongs to the *m* module. Formally, for gene *u* and the *i*th pathway in the *k*th database:

$$\log\frac{Pr\left({\text{gene}}_{u}\in {\text{pathway}}_{i}^{\left(k\right)}\right)}{1-Pr\left({\text{gene}}_{u}\in {\text{pathway}}_{i}^{\left(k\right)}\right)}=b_{i}^{\left(k\right)}+\sum_{m=1}^{M}{\hat{P}}_{um}W_{mi}^{\left(k\right)}$$

A notable advantage of this formulation is the comparability of the $W_{mi}^{\left(k\right)}$ weight values. For each module *m*, the descending order of $W_{m:}^{\left(k\right)}$ values directly corresponds to the relevance of pathways to the module. Conversely, for each pathway *i*, the descending order of $W_{:i}^{\left(k\right)}$ weights mirrors the significance of modules to the pathway’s function.

The nonnegativity constraint on the weight matrices in the final layers has two purposes: Firstly, this serves as a robust regularization technique. Secondly, this formulation is rooted in our 2nd assumption that functional modules are organized into a complex, hierarchical system that encompasses known biological pathways. This constraint reduces the model’s representational capacity to capture only simple additive interactions among modules, disallowing any complex combinations. This formalization can also be seen as a hierarchical model, where modules share a common weight of their genes being annotated with a pathway (i.e. the bias) while also having individual weights. As a result, the model ascertains a positive weight for certain, potentially overlapping modules relevant to a pathway, and it acquires a high negative bias value, rendering all other modules irrelevant.

The objective function minimized during the end-to-end learning of the entire model comprises two parts: the Bernoulli-Poisson loss and the sum of all binary cross-entropy losses of all pathways:

(7) $$L=L_{BP}+\lambda L_{\mathrm{BCE}},$$

where λ is a hyper-parameter that balances the module discovery and interpretational aspects of the two-component model.

### 2.3 Implementation details

Before training, we randomly split the pathways in each database into two disjoint sets: 80% as “training pathways,” which are used intact during training, and 20% as “validation pathways,” utilized separately for training and validation purposes. The purpose of using intact “training pathways” is to provide the model with fully specified pathways to learn from during training. For the training pathways, all pathway-annotated genes serve as ground truth positive data points, while other genes with at least one annotation in the database serve as ground truth negative data points. In the validation pathways, the pathway-annotated genes are further split into a core subset (80%) used as ground truth positive training data points and a validation subset (20%) left out during training, serving as positive validation data points. The other negative genes, not annotated to a specific pathway, are randomly divided (before training) into ground truth negative data points (80%) and negative validation data points (20%). The core subset in the validation pathways serves as a guide for the model to understand which specific pathways it needs to predict during training. Training loss is measured on the ground truth data points from both training and validation pathways, while validation loss is measured on the validation data points, ensuring the model can predict unseen gene-pathway associations. See Supplementary Fig. S2 for an illustration of the train-validation split.

During training, we evaluate the performance every 50 epochs based on the excluded 20% of genes from the validation pathway annotations, using the F1 score. We then compute the mean F1 score across all pathway databases. The models were trained for a maximum of 5000 epochs, with early stopping applied when the mean F1 score did not improve for 10 validation steps. The model that achieved the highest mean F1 score was selected. The mean F1 scores (and standard deviations) for each database and module count setting, averaged over five runs, are shown in Supplementary Table S1.

For each epoch during training, we shuffle all positive edges in the network, divide them into ten equal parts, and perform a training step for each part, using negative sampling (see Section 2.2.1) to sample an equal number of negative edges.

We identified modules with varying module sizes, denoted by *M* (the dimensions of the hidden representations of **F**), specifically using module sizes of 500, 600, 700, 800, 900, and 1000. Our model automatically assigns genes to the identified modules by optimizing the $\tilde{\theta }$ scaling parameter in Equation (4) while we convert community affiliation strengths into module assignment probabilities. We still face a question regarding the appropriate probability cutoff for module assignment. We opt for a natural cutoff of 0.5, where genes with a probability above this threshold are assigned to a module. We conducted an analysis to explore the effect of varying the probability cutoff on the composite scores (see Section 2.4). We evaluated a range of cutoff values from 0.05 to 0.95 in increments of 0.05. As shown in Supplementary Fig. S3, while a cutoff around 0.35 resulted in slightly higher composite scores, the overall trends across different databases and module counts remained consistent. These findings suggest that although the 0.5 cutoff is not optimal, it still performs robustly in most cases. Further details on finding the optimal settings of other hyper-parameters can be found in the Supplementary Methods.

We implemented our method using PyTorch v2.0.1 and PyTorch Geometric v2.3.1. We performed all experiments on a Tesla V100 GPU with 32GB of memory. A single run of GNN4DM takes approximately 1.5–2.5 h, depending on the maximum module count setting, and utilizes 4.2–7.8 GB of GPU memory while training 1.2–2.5 million parameters.

### 2.4 Evaluation

Our evaluation method follows a similar framework to the Disease Module Identification DREAM Challenge, a community effort addressing the problem of disease module identification in complex molecular networks (Choobdar *et al.* 2019). To assess the model’s efficacy in identifying biologically relevant modules, we empirically examined the modules based on their association with complex traits and diseases, leveraging three distinct data sources and using three different computational methods across them to mitigate biases in the assessment:

**GWAS Atlas.** We used gene-level genome-wide association data from the GWAS Atlas project Release 3 (Watanabe *et al.* 2019), specifically the 1211 UK Biobank-specific gene-level summary statistics computed by the MAGMA software. Genes were ranked based on their *P*-values, and for each identified module, a Gene Set Enrichment Analysis using the fgsea R package (Sergushichev 2016) was performed to assess the enrichment of module genes in the rankings.

**FinnGen.** We used GWAS data from the FinnGen project (version DF9, Kurki *et al.* 2023), selecting the top 100 traits by prevalence that could be mapped to level 3 ICD-10 codes. Gene-level summary statistics were computed using the Pascal tool (Lamparter *et al.* 2016). Subsequently, for each module, an aggregated *P*-value was computed with Pascal, which uses a modified Fisher method to determine enrichment in high-scoring genes.

**DisGeNET.** We used all curated gene-disease associations from the DisGeNET database (version 7.0, Piñero *et al.* 2019) and selected diseases with at least 10 associated genes in the network. This resulted in 956 diseases associated with 6326 unique genes. An over-representation analysis was conducted based on a standard hypergeometric statistical test to evaluate the significance of the modules.

Two composite scores were derived to assess a method’s ability to identify biologically meaningful modules:

1. The proportion of traits in each data source for which at least one significant module was identified (assessing the method’s ability to discover at least one disease mechanism for a particular disease).
2. The proportion of modules in each data source for which at least one significant disease/trait was identified (evaluating the method’s capability to ascertain a relevant context for a module in which it is biologically meaningful).

The total score was computed as the summation of the scores from each evaluation data source, with a 5% false discovery rate (FDR) used as the significance threshold.

### 2.5 Baseline methods

We chose several widely used and state-of-the-art community detection algorithms as baseline comparisons for GNN4DM. Utilizing the cdlib python package (Rossetti *et al.* 2019), we identified overlapping and nonoverlapping modules using various commonly used methods (see Supplementary Table S2). Additionally, we used the top three state-of-the-art nonoverlapping module detection algorithms from the DREAM challenge. We also utilized the NOCD and UCoDe GNN-based methods to determine overlapping communities using the same input features as in the case of our method.

We performed an extensive grid search for algorithms with hyper-parameters (see Supplementary Table S2) evaluating them by the modularity density score (Zhang *et al.* 2010). For NOCD and UCoDe, we specified the desired number of modules to match that of our method.

Besides, we also evaluated the pathway databases utilized for fine-tuning the module representations and the three sub-ontologies of the Gene Ontology, treating them as results from a hypothetical overlapping module identification algorithm.

## 3 Results

### 3.1 Identification of modules in the human interactome

We applied the GNN4DM framework on the human interactome derived from the STRING PPI network to identify its overlapping functional modules. The primary variable parameter in our method was the maximum module count, for which we experimented with a range from 500 to 1000 in increments of 100. To evaluate the concordance across these values, we quantified all pairwise overlaps between the various sets of modules and found that the granularity of the modules varies *continuously* by increasing the maximum module count (see Supplementary Results).

We also applied several baseline methods to identify both overlapping and nonoverlapping modules within the network. Comprehensive descriptive statistics for all these methods, alongside the pathway databases used for training GNN4DM, and the Gene Ontology database, are detailed in Supplementary Table S3. The number and average size of modules identified varied significantly across methods, with module counts ranging from 8 (Eigenvector) to 10 965 (Graph Entropy).

GNN4DM and the other two GNN-based methods showed notable differences. NOCD consistently generated far fewer modules than the pre-set maximum, with module counts ranging between 14 and 16. Both NOCD and UCoDe yielded modules with considerably large average sizes (1152–1284 for NOCD, 1359–1612 for UCoDe) but demonstrated low coverage (0.85–0.86 for NOCD, 0.74–0.84 for UCoDe). GNN4DM, on the other hand, consistently produced a module count close to its set maximum, with average module sizes between 72 and 121, and achieved nearly complete coverage (0.99).

As GNN4DM utilizes various pathway databases to refine its internal representations corresponding to the modules, we investigated whether its identified modules simply replicate these pathways or reveal additional information. Our analysis indicated that while GNN4DM aligns with pathway databases, especially with Reactome, it exhibits closer similarity to other methods such as the DREAM methods, CPM, and Graph Entropy, suggesting that GNN4DM identifies modules that are distinct from its training pathways (see Supplementary Results).

### 3.2 Evaluation of functional disease modules

The evaluation of predicted modules poses a challenge due to the absence of a ground truth for “correct” modules against which the identified modules can be compared. To assess the capability of GNN4DM and other methods in identifying biologically relevant *functional disease modules*, we analyzed their associations with complex traits and diseases. This involved using GWAS data from GWAS Atlas and FinnGen, and curated gene-disease associations from DisGeNET. For subsequent analyses, we limited each method’s identified modules to 2–1000 genes. This restriction was based on the assumption that larger modules may lack biological specificity, potentially representing broad cellular functions instead of distinct disease-related processes, or could result from the method’s inability to cluster a large group of genes into smaller sub-modules (see Supplementary Table S4 for descriptive statistics after filtering).

**Figure 2.**
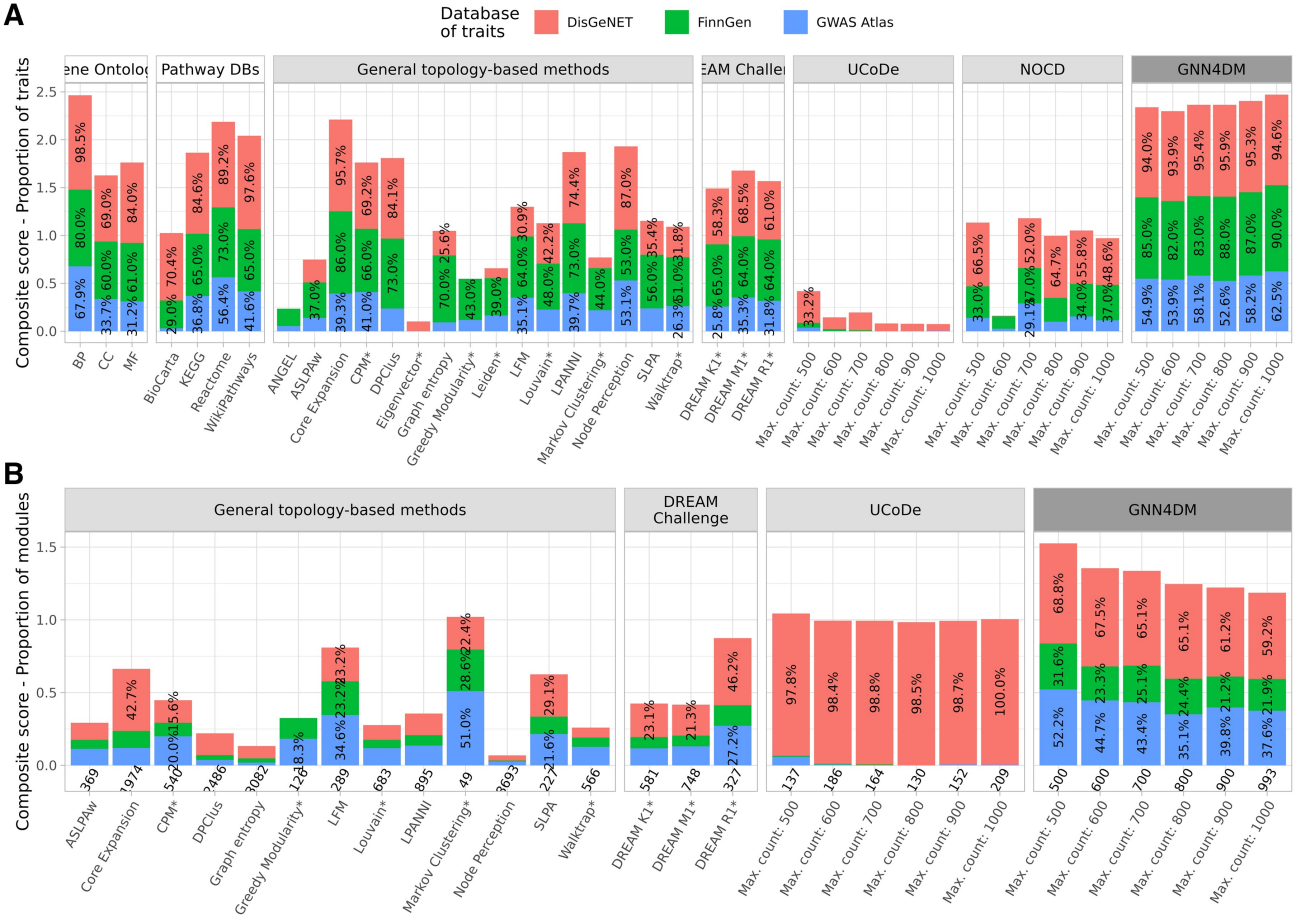
Performance evaluation of module identification methods and pathway databases. (A) Composite scores defined by the cumulative proportion of traits for which at least one significant module was identified across each data source. (B) Composite scores defined by the cumulative proportion of modules for which at least one significant trait was identified across each data source. Values under the columns indicate the number of modules in the size range of 2–1000. For part B, pathway databases and methods that identified fewer than 25 modules are omitted. Column groups with a white background header color indicate biological databases (in A), light gray indicates baseline methods, and dark gray indicates GNN4DM (on A&B). Percentage values inside the columns show the proportion of traits (in A), or modules (in B) that are significantly enriched/over-represented in the corresponding trait data source. Only percentages >25% (on A) or 15% (on B) are shown. Method names denoted with an asterisk correspond to nonoverlapping module identification.

First, we calculated a total score for each method, defined as the cumulative proportion of traits for which at least one significant module was identified. Our method, GNN4DM, outperformed all other methods in this regard, regardless of the maximum module count set within it (see Fig. 2A). The highest score was observed with 1000 modules (total score = 2.47), surpassing even the scores of pathway databases used in training (ranging from 1.02 to 2.19). This score was also higher than, or on par with, the score achieved by Gene Ontology (2.46), which was not part of our training set. Specifically, GNN4DM with 1000 modules identified significant modules for the highest proportion of traits in the GWAS Atlas (62.5%), exceeding the next best method (Node Perception, 53.1%) by 9.4 percentage points, and in FinnGen (90%), outperforming the second-best (Core Expansion, 86%) by 4 percentage points. For DisGeNET, GNN4DM identified at least one significant module for 94.6% of diseases, closely matching the top-performing method (Core Expansion, 95.7%). On average, across all module size settings, GNN4DM discovered at least one significant module for 94.9% of traits in DisGeNET, 85.8% of diseases in FinnGen, and 56.7% of traits in GWAS Atlas.

Next, we computed another total score for each method, defined as the cumulative proportion of modules associated with at least one disease or trait. This score quantifies a method’s ability to discover a relevant context for their modules in which these are biologically meaningful. For this analysis, we omitted pathway databases to avoid selection bias, and methods that identified fewer than 25 modules to avoid bias due to low module counts. Again, GNN4DM consistently surpassed all other methods, regardless of its maximum module count setting (see Fig. 2B), demonstrating a high proportion of significant modules across all three trait datasets. The relatively high proportion of significantly associated modules with respect to DisGeNET traits observed in UCoDe is due to the fact that many of the modules discovered by this method were nearly identical. That is, one module had many slightly different copies, and all variants of this module were associated with at least one DisGeNET trait. As anticipated, the proportion of significant modules in GNN4DM is inversely correlated with its maximum module count. Across all module size settings, 64.5% of modules identified by GNN4DM were associated with at least one trait in DisGeNET, 24.6% in FinnGen, and 42.1% in GWAS Atlas.

The same qualitative trends hold when using more stringent significance thresholds (see Supplementary Figs S4 and S5). Overall, these findings underscore GNN4DM’s effectiveness in detecting a relatively high proportion of modules associated with a broad spectrum of diseases and complex traits.

### 3.3 Interpretation of two highly enriched disease modules

We selected and visualized two highly enriched modules to demonstrate the interpretation of modules identified by GNN4DM, highlighting their associated diseases and relevant pathways.

#### 3.3.1 Module 1

A notably enriched multimorbidity module (see Supplementary Fig. S6A), derived using a maximum module count of 500, showed significant enrichment across the trait datasets: 39% of the diseases in the FinnGen dataset (39 out of the 100 most prevalent diseases with 3-level ICD codes), 11.8% in GWAS Atlas traits (143 out of 1211), and in a lesser extent, 2.82% in DisGeNET disorders (27 out of 956). This module is composed of four highly interconnected sub-parts, containing: (i) histone-coding genes, including core histones (H2A, H2B, H3, H4) and the H1 linker histone, crucial for DNA packaging and gene expression regulation. Also, in their extranuclear form, these histones are known to enhance host defense functions and contribute to inflammatory responses (Li *et al.* 2022); (ii) the Human Leukocyte Antigen complex, corresponding to MHC class II (DP, DM, DO, DQ, DR), which plays an essential role in the immune response against extracellular pathogens; (iii) the CENP-A NAC/CAD kinetochore complex, vital for chromosome segregation during cell division, thereby maintaining genetic stability; and (iv) cytokine-coding genes, involved in Th1 and Th2 immune responses, acting as key modulators of the body’s immune reaction.

To further understand the module’s biological relevance, we inspected the weights GNN4DM learned for the module in association with the known biological pathways. The module’s strong relation to immune-related pathways, including Th1/Th2, CTLA4, IL-4, and NF-kB signaling, emphasizes its role in immune regulation. Additionally, its association with pathways related to chromosome maintenance, gene expression regulation, and cellular senescence highlights its broader involvement in genetic stability and cellular aging processes (see Supplementary Fig. S6C). This is also reflected by the module’s disease associations (see Supplementary Fig. S6B) with various immunological disorders (like type 1 diabetes, asthma, celiac disease, allergic rhinitis, and eczema) and conditions tied to genomic instability (such as malignancies and schizophrenia).

#### 3.3.2 Module 2

**Figure 3.**
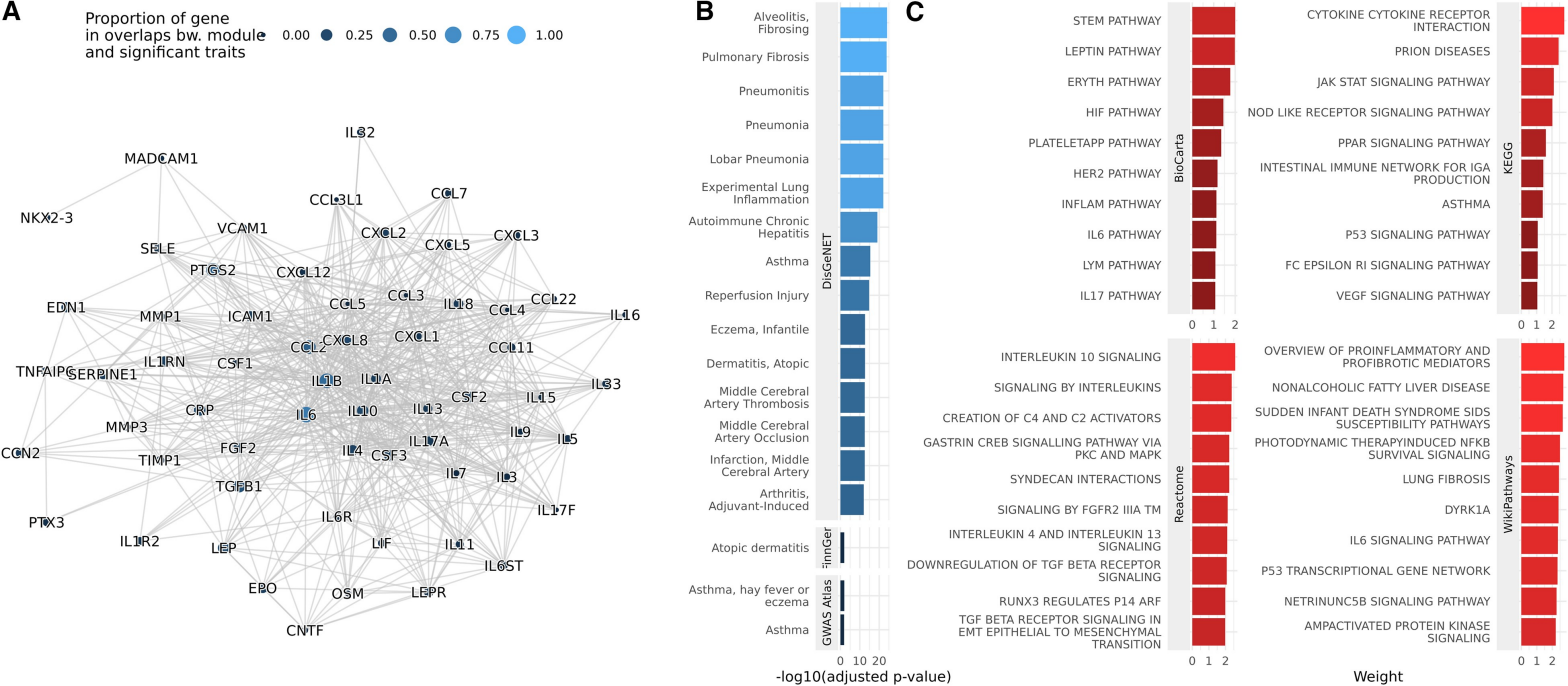
A multimorbidity module significantly enriched in multiple diseases. (A) The graph displays a subnetwork of the STRING PPI network, representing the module. The size and color of each node denote the gene’s proportion among disease-associated genes in those diseases that show significant overlap with the module, as determined by DisGeNET. (B) The top (at most) 15 diseases significantly associated with the module, as identified in the 3 evaluation datasets. (C) The top 10 pathways in each pathway database that are relevant to the module. The bars show the weight GNN4DM learned for the module in association with the corresponding pathway.

Another module highly over-represented in 17.4% among DisGeNET’s disorders (166 out of 956) is shown in Fig. 3A. This module is comprised of a single densely interconnected component, primarily formed by cytokines, including chemokines from the CC and CXC sub-families, several interleukins, cytokine receptors, various regulatory protein-coding genes, and several other genes. The genes within the module are predominantly involved in immune response processes, inflammation, and related pathomechanisms. Notably, it encompasses both pro-inflammatory (e.g. *IL1B*, *IL18*) and anti-inflammatory (e.g. *IL10*, *IL4*) cytokine-coding genes, as well as interleukins with dual roles (e.g. *IL6*), suggesting a function in regulating the balance between these contrasting immune processes.

The potential functions of the module are also reflected in its related pathways, which include the Cytokines and inflammatory response pathway, the Cytokine-cytokine receptor interaction pathway, and several interleukin signaling pathways, among others (see Fig. 3C). The associated diseases include several lung diseases, such as pneumonia, pulmonary fibrosis, and asthma; autoimmune disorders, such as rheumatoid arthritis and autoimmune chronic hepatitis; inflammatory diseases, such as atopic dermatitis and eczema; and various others (see Fig. 3B, and Supplementary Fig. S7).

## 4 Conclusion

In this study, we introduced GNN4DM, a novel graph neural network-based approach for identifying functional disease modules in PPI networks. GNN4DM has demonstrated strong capability in detecting biologically meaningful modules by integrating a substantial amount of genomic data and aligning the modules with known biological pathways in an end-to-end manner. Our method not only outperforms existing techniques in module identification but also provides valuable interpretations by linking modules to specific pathways. GNN4DM’s case studies further illustrate its utility in revealing complex disease mechanisms. Overall, GNN4DM advances the understanding of complex diseases by providing a two-component model that integrates a crucial part of the functional disease module discovery pipeline.

## Supplementary Material

btae573_Supplementary_Data

## Data Availability

The data underlying this article are available in GitHub, at https://github.com/gezsi/gnn4dm. The datasets were derived from sources in the public domain: STRING, https://string-db.org/; MSigDB, https://www.gsea-msigdb.org/gsea/msigdb/; GTExPortal, https://gtexportal.org/home/; GWASATLAS, https://atlas.ctglab.nl/.

## References

[btae573-B1] Aguet F , Anand S, Ardlie KG et al The GTEx consortium atlas of genetic regulatory effects across human tissues. Science 2020;369:1318–30. 32913098 10.1126/science.aaz1776 PMC7737656

[btae573-B2] Chagoyen M , Pazos F. Characterization of clinical signs in the human interactome. Bioinformatics 2016;32:1761–5. 26861820 10.1093/bioinformatics/btw054

[btae573-B3] Chen J , Gong Z, Mo J et al Self-training enhanced: network embedding and overlapping community detection with adversarial learning. IEEE Trans Neural Netw Learn Syst 2022;33:6737–48. 34111000 10.1109/TNNLS.2021.3083318

[btae573-B4] Choobdar S , Ahsen ME, Crawford J et al; DREAM Module Identification Challenge Consortium. Assessment of network module identification across complex diseases. Nat Methods 2019;16:843–52. 31471613 10.1038/s41592-019-0509-5 PMC6719725

[btae573-B5] Clune J , Mouret JB, Lipson H. The evolutionary origins of modularity. Proc Biol Sci 2013;280:20122863. 23363632 10.1098/rspb.2012.2863 PMC3574393

[btae573-B6] Gavin A-C , Bösche M, Krause R et al Functional organization of the yeast proteome by systematic analysis of protein complexes. Nature 2002;415:141–7. 11805826 10.1038/415141a

[btae573-B7] Hagberg AA , Schult DA, Swart PJ. Exploring network structure, dynamics, and function using networkx. In: Varoquaux G, Vaught T, Millman J (eds.), *Proceedings of the 7th Python in Science Conference*, Pasadena, CA, USA, 2008, 11–5.

[btae573-B8] Hartwell LH , Hopfield JJ, Leibler S et al From molecular to modular cell biology. Nature 1999;402:C47–52. 10591225 10.1038/35011540

[btae573-B9] Hatleberg WL , Hinman VF. Chapter two—modularity and hierarchy in biological systems: using gene regulatory networks to understand evolutionary change. In: Gilbert SF (ed.), Evolutionary Developmental Biology, volume 141 of Current Topics in Developmental Biology. Academic Press, 2021, 39–73. 10.1016/bs.ctdb.2020.11.004 33602494

[btae573-B10] Huang JK , Carlin DE, Yu MK et al Systematic evaluation of molecular networks for discovery of disease genes. Cell Syst 2018;6:484–95.e5. 29605183 10.1016/j.cels.2018.03.001 PMC5920724

[btae573-B11] Jia Y, Zhang Q, Zhang W et al Communitygan: Community detection with generative adversarial nets. In: *The Web Conference 2019—Proceedings of the World Wide Web Conference, WWW 2019*. San Francisco, CA: Association for Computing Machinery, New York, NY, 2019, 784–94.

[btae573-B12] Kipf TN , Welling M. Semi-supervised classification with graph convolutional networks. In *Proceedings of the 5th International Conference on Learning Representations*, ICLR’17, 2017.

[btae573-B13] Kurki MI , Karjalainen J, Palta P et al; FinnGen. Finngen provides genetic insights from a well-phenotyped isolated population. Nature 2023;613:508–18. 36653562 10.1038/s41586-022-05473-8 PMC9849126

[btae573-B14] Lamparter D , Marbach D, Rueedi R et al Fast and rigorous computation of gene and pathway scores from SNP-based summary statistics. PLoS Comput Biol 2016;12:e1004714. 26808494 10.1371/journal.pcbi.1004714 PMC4726509

[btae573-B15] Li H , Han Z, Sun Y et al CGMEGA: explainable graph neural network framework with attention mechanisms for cancer gene module dissection. Nat Commun 2024;15:5997. 39013885 10.1038/s41467-024-50426-6 PMC11252405

[btae573-B16] Li X , Ye Y, Peng K et al Histones: the critical players in innate immunity. Front Immunol 2022;13:1030610. 36479112 10.3389/fimmu.2022.1030610 PMC9720293

[btae573-B17] Loscalzo J , Barabasi AL. Systems biology and the future of medicine. Wiley Interdiscip Rev Syst Biol Med 2011;3:619–27. 21928407 10.1002/wsbm.144 PMC3188693

[btae573-B18] Manipur I , Giordano M, Piccirillo M et al Community detection in protein–protein interaction networks and applications. IEEE/ACM Trans Comput Biol Bioinform 2023;20:217–37. 34951849 10.1109/TCBB.2021.3138142

[btae573-B19] Manners HN , Roy S, Kalita JK. Intrinsic-overlapping co-expression module detection with application to Alzheimer’s disease. Comput Biol Chem 2018;77:373–89. 30466046 10.1016/j.compbiolchem.2018.10.014

[btae573-B20] Menche J , Sharma A, Kitsak M et al Uncovering disease–disease relationships through the incomplete interactome. Science 2015;347:1257601. 25700523 10.1126/science.1257601 PMC4435741

[btae573-B21] Mengistu H , Huizinga J, Mouret J-B et al The evolutionary origins of hierarchy. PLoS Comput Biol 2016;12:e1004829. 27280881 10.1371/journal.pcbi.1004829 PMC4900613

[btae573-B22] Mitra K , Carvunis A-R, Ramesh SK et al Integrative approaches for finding modular structure in biological networks. Nat Rev Genet 2013;14:719–32. 24045689 10.1038/nrg3552 PMC3940161

[btae573-B23] Moradan A , Draganov A, Mottin D et al Ucode: unified community detection with graph convolutional networks. Mach Learn 2023;112:5057–80.

[btae573-B24] Ni P , Wang J, Zhong P et al Constructing disease similarity networks based on disease module theory. IEEE/ACM Trans Comput Biol Bioinform 2020;17:906–15. 29993782 10.1109/TCBB.2018.2817624

[btae573-B25] Oti M , Brunner H. The modular nature of genetic diseases. Clin Genet 2007;71:1–11. 17204041 10.1111/j.1399-0004.2006.00708.x

[btae573-B26] Palla G , Derényi I, Farkas I et al Uncovering the overlapping community structure of complex networks in nature and society. Nature 2005;435:814–8. 15944704 10.1038/nature03607

[btae573-B27] Pfeifer B , Saranti A, Holzinger A. GNN-SubNet: disease subnetwork detection with explainable graph neural networks. Bioinformatics 2022;38:ii120–6. 36124793 10.1093/bioinformatics/btac478

[btae573-B28] Pfeifer B , Chereda H, Martin R et al Ensemble-GNN: federated ensemble learning with graph neural networks for disease module discovery and classification. Bioinformatics 2023;39:btad703. 37988152 10.1093/bioinformatics/btad703 PMC10684359

[btae573-B29] Piñero J , Ramírez-Anguita JM, Saüch-Pitarch J et al The disGeNET knowledge platform for disease genomics: 2019 update. Nucleic Acids Res 2019;48:D845–55. 10.1093/nar/gkz1021 PMC7145631 31680165

[btae573-B30] Psorakis I , Roberts S, Ebden M et al Overlapping community detection using Bayesian non-negative matrix factorization. Phys Rev E Stat Nonlin Soft Matter Phys 2011;83:066114. 21797448 10.1103/PhysRevE.83.066114

[btae573-B31] Ravasz E , Somera AL, Mongru DA et al Hierarchical organization of modularity in metabolic networks. Science 2002;297:1551–5. 12202830 10.1126/science.1073374

[btae573-B32] Rossetti G , Milli L, Cazabet R. CDLIB: a python library to extract, compare and evaluate communities from complex networks. Appl Netw Sci 2019;4:52.

[btae573-B33] Sergushichev AA. An algorithm for fast preranked gene set enrichment analysis using cumulative statistic calculation. bioRxiv, 10.1101/060012, 2016, preprint: not peer reviewed.

[btae573-B34] Sharan R , Ulitsky I, Shamir R. Network‐based prediction of protein function. Mol Syst Biol 2007;3:88. 17353930 10.1038/msb4100129 PMC1847944

[btae573-B35] Shchur O , Günnemann S. Overlapping community detection with graph neural networks. arXiv, https://arxiv.org/abs/1909.12201, 2019, preprint: not peer reviewed.

[btae573-B36] Soyer OS. Emergence and maintenance of functional modules in signaling pathways. BMC Evol Biol 2007;7:205. 17974002 10.1186/1471-2148-7-205 PMC2228312

[btae573-B37] Subramanian A , Tamayo P, Mootha VK et al Gene set enrichment analysis: a knowledge-based approach for interpreting genome-wide expression profiles. Proc Natl Acad Sci USA 2005;102:15545–50. 16199517 10.1073/pnas.0506580102 PMC1239896

[btae573-B38] Szklarczyk D , Gable AL, Lyon D et al String v11: protein–protein association networks with increased coverage, supporting functional discovery in genome-wide experimental datasets. Nucleic Acids Res 2019;47:D607–13. 30476243 10.1093/nar/gky1131 PMC6323986

[btae573-B39] Wang T , Peng Q, Liu B et al Disease module identification based on representation learning of complex networks integrated from GWAS, EQTL summaries, and human interactome. Front Bioeng Biotechnol 2020;8:418. 32435638 10.3389/fbioe.2020.00418 PMC7218106

[btae573-B40] Watanabe K , Stringer S, Frei O et al A global overview of pleiotropy and genetic architecture in complex traits. Nat Genet 2019;51:1339–48. 31427789 10.1038/s41588-019-0481-0

[btae573-B41] Yang J , Leskovec J. Overlapping community detection at scale: A nonnegative matrix factorization approach. In: *Proceedings of the Sixth ACM International Conference on Web Search and Data Mining, WSDM ’13*. New York, NY: Association for Computing Machinery, 2013, 587–96.

[btae573-B42] Ye X , Wu Y, Pi J et al Deepgmd: a graph-neural-network-based method to detect gene regulator module. IEEE/ACM Trans Comput Biol Bioinform 2022;19:3366–73. 34546926 10.1109/TCBB.2021.3114281

[btae573-B43] Yuan S , Zeng H, Zuo Z et al Overlapping community detection on complex networks with graph convolutional networks. Comput Commun 2023;199:62–71.

[btae573-B44] Zhang S , Ning X-M, Ding C et al Determining modular organization of protein interaction networks by maximizing modularity density. BMC Syst Biol 2010;4:S10. 20840724 10.1186/1752-0509-4-S2-S10 PMC2982684

[btae573-B45] Zhang Y, Xiong Y, Ye Y et al Seal: learning heuristics for community detection with generative adversarial networks. In: *Proceedings of the 26th ACM SIGKDD International Conference on Knowledge Discovery and Data Mining, KDD '20*. New York, NY: Association for Computing Machinery, 2020, 1103–13.

[btae573-B46] Zhao S , Li S. A co-module approach for elucidating drug–disease associations and revealing their molecular basis. Bioinformatics 2012;28:955–61. 22285830 10.1093/bioinformatics/bts057

