# Supplementary material for: GNN4DM: a graph neural network-based method to identify overlapping functional disease modules
Source: Bioinformatics. 2024 Sep 25;40(10):btae573. doi: 10.1093/bioinformatics/btae573 (PMC11639141; doi:10.1093/bioinformatics/btae573)
Supplement: btae573_Supplementary_Data [file btae573_supplementary_data.pdf]

Supplementary Material for GNN4DM: A Graph Neural Network-based method to identify overlapping functional disease modules

András Gézsi, Péter Antal
Department of Artificial Intelligence and Systems Engineering,
Budapest University of Technology and Economics, Budapest, Hungary
gezsi.andras@vik.bme.hu
antal.peter@vik.bme.hu

1 Supplementary Results

1.1 Concordance of GNN4DM across various maximum module count settings

The primary variable parameter in GNN4DM was the maximum module count, for which we experimented with a range from 500 to 1000 in increments of 100. To evaluate the concordance across these values, we quantified all pairwise overlaps between the various sets of modules by categorizing them as strong, sub-module, or partial (see Supplementary Methods). Our analysis indicated a substantial agreement among the chosen module counts, predominantly characterized by a high percentage of partial overlaps (see Supplementary Figure S8). Besides, increasing the maximum module count parameter tended to yield smaller sub-modules nested within those identified at lower counts. These observations, together with the generally low percentage of strong overlaps, suggest that the granularity of the modules varies *continuously* by increasing the maximum module count from larger, broader modules down to smaller, more specific subsets. This is also evident by the inverse relationship between the average module size and the maximum module count (see Supplementary Table S3).

1.2 Similarity and complementarity of different module identification methods

As GNN4DM utilizes various pathway databases to refine its internal representations corresponding to the modules, a relevant question arises: do the modules identified by GNN4DM merely replicate these pathways, or do they capture extra layers of information? To address this, we conducted two analyses.

Initially, we measured all pairwise overlaps between GNN4DM-identified modules and pathways in the databases, classifying them as strong, sub-module, or partial overlaps (see Supplementary Methods). We observed a generally low rate of strong overlap, with less than 2% of GNN4DM modules closely matching any pathway from the databases. Conversely, partial overlaps were more common (see Supplementary Figure S9A). Meanwhile, the pathways often tend to be sub-modules of larger GNN4DM modules, suggesting that these modules represent compact, higher-level biological functions that encompass multiple known pathways (see Supplementary Figure S9B).

Subsequently, to discern whether different methods reveal similar or distinct modules, we calculated a pairwise similarity metric among module identification methods and visualized their relationships using multidimensional scaling (see Supplementary Figure S10 and Supplementary Methods) and a heatmap combined with hierarchical clustering (see Supplementary Figure S11). This analysis demonstrated that although GNN4DM variants show alignment with pathway databases, particularly Reactome, they exhibit closer similarity to other approaches, including DREAM methods, CPM, and Graph Entropy. This underscores that GNN4DM identifies modules distinct from the pathways used for training it.

1.3 Hierarchical structure, similarity and complementarity of pathway databases

We characterized all pairwise overlaps between the pathways in each pathway database using the same overlap definitions as in the previous sections (i.e. by categorizing them as strong, sub-module, or partial overlaps; see Supplementary Methods). Our analysis revealed that Reactome exhibits a more extensive hierarchical structure compared to the other databases, as indicated by the high proportion of strongly overlapping pathways within itself (see Supplementary Figure S12). This analysis also showed that the pathway databases contain complementary information or pathways at various granularity levels. This can also be seen in Supplementary Table S3, which details the average number of genes in the pathways. BioCarta contains highly granular modules (average size: 19), while the other three databases have lower granularity levels (average sizes: 47.4-66). Our results further indicated that a higher proportion of BioCarta pathways are sub-

modules of pathways in other databases (see first row in Supplementary Figure S12). Additionally, many pathways in each database are sub-modules of Reactome pathways (see third column in Supplementary Figure S12). Leveraging these diverse databases ensures that the module identification in the GNN4DM model is guided by a wide range of biological information, from more specific functional modules to broader pathways.

1.4 Enrichment of modules across the three trait datasets

To conduct a more in-depth analysis of modules identified by GNN4DM and their associations with traits, we generated pairwise scatter plots showing the proportion of traits associated with these modules in the trait datasets (see Supplementary Figure S13). A significant correlation is observed between GWAS Atlas and FinnGen, indicating that modules highly enriched in one database tend to be similarly enriched in the other (see Supplementary Figure S13A). However, the correlation between DisGeNET and the GWAS databases is markedly lower, or in some cases, even negative (see Supplementary Figures S13B and C). This discrepancy could be attributed to the differing disease prevalences across the datasets, particularly since DisGeNET includes a larger proportion of rare diseases. Additionally, the over-representation analysis used for DisGeNET tends to favor larger gene sets (as the mean number of associated genes is also larger in this dataset), unlike the enrichment methods applied to the other two databases.

2 Supplementary Methods

2.1 Implementation details

2.1.1 Model performance

The F1 scores achieved during training for the validation pathways were not reported in the main text, as they were not our primary focus. Our main objective was to empirically assess module identification based on their association with complex traits and diseases, given the lack of a definitive ground truth. However, for completeness, we present the mean F1 scores (and standard deviations) for each database and module count setting, with the train-validation split applied in each run. These results are averaged over five runs and are shown in Supplementary Table S1.

2.1.2 Hyper-parameter optimalization

We conducted exploratory experiments to find the optimal settings of other hyper-parameters, including the learning rate, the used learning rate scheduler and its hyper-parameters, the number of hidden GCN layers, the number of neurons, and the λ hyper-parameter weighing the BCE loss component, among others. We also experimented with various graph neural network architectures, including Graph Attention layers and GraphSAGE, alongside different regularization techniques like dropout, and L1 or L2 regularization of the weight matrices. However, these alterations did not yield any noticeable improvements. Additionally, we attempted to incorporate the Gene Ontology database as target outputs for the model, yet this unexpectedly led to inferior results.

We employed the AdamW optimizer with a starting learning rate of 0.001 and a Step learning rate scheduler, which decays the learning rate for each parameter group by 0.85 every 250 epochs.

We show the finalized hyper-parameters for our model in Supplementary Table S5.

2.2 A note on the Bernoulli-Poisson model

The rationale behind Eq. 2 in the main paper stems from our first assumption, namely: (1.a) Each gene u belongs to a given functional module m with a given strength F_{um}. The higher the weight is, the more likely that this gene interacts with other members of the same module, depending on their own weights. (1.b) A gene (or a gene product) may have various functional roles in the cell, which are carried out by different functional modules. This way, a gene may belong to multiple modules and, therefore, has multiple independent chances to interact with another gene v if they both participate in multiple modules. In other words, the more modules two genes share, the higher the probability of their interaction (i.e., an edge appears in the graph). More formally, we assume that a

pair of genes (u, v) has a latent interaction of strength $S_{uv}^{(m)}$ within the m module, and this strength variable has a Poisson distribution with mean $F_{um} \cdot F_{vm}$. Then, the aggregate interaction effect, denoted as S_{uv}, between nodes u and v, is obtained by summing over all m modules, namely $S_{uv} = \sum_m S_{uv}^{(m)}$. Then, according to the additivity rule of Poisson distributed random variables, $S_{uv} \sim \text{Pois}(\mathbf{f}_u \mathbf{f}_v^\top)$, where $\mathbf{f}_u = F_{u:}$ and $\mathbf{f}_v = F_{v:}$. The probability of the edge between (u, v) amounts to the probability of S_{uv} being non-zero, i.e.: $P(S_{uv} > 0) = 1 - P(S_{uv} = 0) = 1 - exp(-\mathbf{f}_u \mathbf{f}_v^\top)$, which is the same as Eq. 2.

2.3 Similarity of module predictions

Adapting the approach from [3], we defined a similarity measure for comparing module predictions from different methods. We represented the set of modules from a method m as a prediction vector P_m, with a length of $N(N-1)/2$, where N represents the total number of genes in the network. Each vector element corresponds to a gene pair, denoting the count of modules encompassing both genes. To quantify the similarity between two methods $m1$ and $m2$, we computed their distance as:

$$D(m_1, m_2) = 1 - \frac{< P_{m1}, P_{m2} >}{||P_{m1}||_2 \cdot ||P_{m2}||_2},$$

where $< ., . >$ is the inner product and $||.||_2$ is the Euclidean norm. The resulting symmetric distance matrix D served as input for multidimensional scaling (MDS) to reduce dimensionality in Supplementary Figure S10. This distance matrix was also visualized as a heatmap in Supplementary Figure S11. This metric extends the approach of [3] for non-overlapping modules to overlapping ones.

2.4 Overlap between trait-associated modules

In line with [3], we employed various metrics to quantify the overlap between modules from various methods. First, we evaluated the statistical significance of the overlaps using hypergeometric distribution. An overlap was deemed statistically significant if its Bonferroni-adjusted p-value was below 0.05, taking into account the total number of module pair comparisons conducted. Next, we quantified the overlap using the Jaccard index, which is the ratio of the size of the intersection to the size of the union of two modules (i.e., gene sets) A and B:

$$J(A, B) = \frac{|A \cap B|}{|A \cup B|}$$

Additionally, to identify sub-modules, we also considered the proportion of genes in the first module present in the second module:

$$S(A, B) = \frac{|A \cap B|}{|A|}$$

Based on these metrics, we classified each statistically significant overlap between module A and another module B into three categories:

1. Strong overlap: $J(A, B) \geq 0.5$.
2. Sub-module: $J(A, B) < 0.5$ and $S(A, B) - J(A, B) \geq 0.5$.
3. Partial overlap: $J(A, B) < 0.5$ and $S(A, B) - J(A, B) < 0.5$.

We applied these classifications to get an overview of the granularity of different maximum module count settings in GNN4DM, and to compare the modules from GNN4DM with all pathways of the pathway databases utilized for training (see Supplementary Figures S8 and S9).

3 Supplementary Tables

Table S1: F1 score (and the standard deviation) performance metric for different module counts across pathway databases, averaged over 5 runs.

Module count	BioCarta	KEGG	Reactome	WikiPathways
500	0.577 (0.009)	0.762 (0.006)	0.623 (0.026)	0.422 (0.01)
600	0.584 (0.011)	0.776 (0.009)	0.633 (0.01)	0.423 (0.012)
700	0.582 (0.009)	0.771 (0.014)	0.646 (0.015)	0.429 (0.005)
800	0.57 (0.015)	0.777 (0.009)	0.649 (0.018)	0.42 (0.011)
900	0.565 (0.012)	0.776 (0.008)	0.656 (0.009)	0.431 (0.004)
1000	0.592 (0.01)	0.784 (0.011)	0.652 (0.01)	0.427 (0.005)

Table S2: Hyper-parameterization and references of baseline methods.

<table>
<tr><th></th><th>Method</th><th>Overlaps</th><th>Hyper-parameters</th><th>Reference</th></tr>
<tr><td rowspan="16">General topology-based methods</td><td>CPM</td><td>✗</td><td>Resolution: from 0.01 to 0.1 by 0.01</td><td>[15]</td></tr>
<tr><td>Eigenvector</td><td>✗</td><td>None</td><td>[10]</td></tr>
<tr><td>Greedy Modularity</td><td>✗</td><td>None</td><td>[5]</td></tr>
<tr><td>Leiden</td><td>✗</td><td>None</td><td>[16]</td></tr>
<tr><td>Louvain</td><td>✗</td><td>Resolution: from 0.1 to 1.0 by 0.1
Randomize: True</td><td>[2]</td></tr>
<tr><td>Markov Clustering</td><td>✗</td><td>Inflation: from 1.1 to 1.8 by 0.1</td><td>[6]</td></tr>
<tr><td>Walktrap</td><td>✗</td><td>None</td><td>[11]</td></tr>
<tr><td>ANGEL</td><td>✓</td><td>Threshold: from 0.1 to 1.0 by 0.1</td><td>[12]</td></tr>
<tr><td>ASLPAw</td><td>✓</td><td>None</td><td>[17]</td></tr>
<tr><td>Core Expansion</td><td>✓</td><td>None</td><td>[4]</td></tr>
<tr><td>DPClus</td><td>✓</td><td>Density th.: from 0.8 to 0.95 by 0.05
Property th.: from 0.3 to 0.7 by 0.1</td><td>[1]</td></tr>
<tr><td>Graph entropy</td><td>✓</td><td>None</td><td>[7]</td></tr>
<tr><td>LFM</td><td>✓</td><td>Alpha: from 0.5 to 2.0 by 0.1</td><td>[8]</td></tr>
<tr><td>LPANNI</td><td>✓</td><td>Threshold: 0.01</td><td>[9]</td></tr>
<tr><td>Node Perception</td><td>✓</td><td>Threshold: from 0.2 to 0.8 by 0.2
Overlap th.: from 0.2 to 0.8 by 0.2</td><td>[13]</td></tr>
<tr><td>SLPA</td><td>✓</td><td>R: from 0.1 to 0.5 by 0.1</td><td>[17]</td></tr>
<tr><td rowspan="3">DREAM Challenge</td><td>DREAM K1</td><td>✗</td><td>None</td><td rowspan="3">[3, 14]</td></tr>
<tr><td>DREAM M1</td><td>✗</td><td>None</td></tr>
<tr><td>DREAM R1</td><td>✗</td><td>None</td></tr>
</table>

Table S3: Descriptive statistics for the investigated module identification methods and pathway databases, unfiltered.

	Method	Overlaps	Count of identified modules	Average size (range)	Node coverage	Mean number of modules per gene	Conductance	Modularity overlap
General topology-based methods	CPM	✗	1583	10.0 (1 - 830)	1.00	1.00	0.83	0.03
	Eigenvector	✗	8	1974.1 (845 - 3877)	1.00	1.00	0.27	0.01
	Greedy Modularity	✗	130	121.5 (2 - 5611)	1.00	1.00	0.35	0.27
	Leiden	✗	32	493.5 (5 - 1693)	1.00	1.00	0.19	0.15
	Louvain	✗	686	23.0 (2 - 1577)	1.00	1.00	0.47	0.13
	Markov Clustering	✗	52	303.7 (2 - 6990)	1.00	1.00	0.41	0.10
	Walktrap	✗	899	17.6 (1 - 4656)	1.00	1.00	0.64	0.13
	ANGEL	✓	17	762.1 (4 - 12790)	0.82	1.00	0.16	0.51
	ASLPAw	✓	583	29.6 (1 - 6253)	1.00	1.09	0.67	0.08
	Core Expansion	✓	1974	20.5 (2 - 276)	0.82	3.12	0.81	0.06
	DPClus	✓	2486	6.0 (2 - 185)	0.77	1.22	0.78	-0.21
	Graph entropy	✓	10965	2.4 (1 - 198)	1.00	1.66	0.95	-0.07
	LFM	✓	291	100.2 (2 - 13988)	1.00	1.85	0.44	0.07
	LPANNI	✓	899	24.2 (1 - 3233)	1.00	1.38	0.56	0.15
	Node Perception	✓	8699	6.7 (2 - 2183)	0.95	3.90	0.94	-0.09
	SLPA	✓	267	55.6 (1 - 2523)	0.94	1.00	0.53	0.09
DREAM Challenge	DREAM K1	✗	581	27.2 (2 - 100)	1.00	1.00	0.59	0.08
	DREAM M1	✗	2029	7.8 (1 - 100)	1.00	1.00	0.85	-0.02
	DREAM R1	✗	327	44.7 (3 - 93)	0.93	1.00	0.61	0.04
UCoDe	Max. count: 500	✓	498	1359.2 (5 - 2317)	0.74	57.57	0.23	0.00
	Max. count: 600	✓	599	1413.0 (12 - 2669)	0.77	69.73	0.22	0.00
	Max. count: 700	✓	698	1521.7 (5 - 2955)	0.77	87.37	0.21	0.00
	Max. count: 800	✓	800	1591.3 (2 - 2946)	0.80	100.70	0.21	0.00
	Max. count: 900	✓	899	1582.4 (9 - 3436)	0.82	109.49	0.21	0.00
	Max. count: 1000	✓	999	1611.5 (82 - 3287)	0.84	121.14	0.22	0.00
NOCD	Max. count: 500	✓	15	1199.7 (437 - 2240)	0.85	1.33	0.35	0.02
	Max. count: 600	✓	16	1152.0 (1 - 2226)	0.86	1.36	0.39	0.01
	Max. count: 700	✓	15	1215.5 (352 - 2315)	0.86	1.34	0.34	0.02
	Max. count: 800	✓	14	1277.8 (13 - 2231)	0.85	1.33	0.37	0.01
	Max. count: 900	✓	14	1283.6 (39 - 2225)	0.85	1.33	0.33	0.02
	Max. count: 1000	✓	15	1180.7 (7 - 2431)	0.85	1.32	0.35	0.01
**GNN4DM**	Max. count: 500	✓	500	120.8 (6 - 659)	0.99	3.86	0.72	-0.00
	Max. count: 600	✓	600	102.3 (4 - 603)	0.99	3.91	0.73	-0.00
	Max. count: 700	✓	700	95.4 (4 - 527)	0.99	4.26	0.73	-0.00
	Max. count: 800	✓	800	81.8 (4 - 666)	0.99	4.17	0.75	-0.01
	Max. count: 900	✓	900	75.0 (2 - 462)	0.99	4.30	0.76	-0.01
	Max. count: 1000	✓	993	72.3 (3 - 544)	0.99	4.57	0.76	-0.01
Pathways	BioCarta	✓	221	19.0 (10 - 78)	0.08	3.17	0.94	-0.10
	KEGG	✓	186	66.0 (10 - 321)	0.31	2.51	0.77	-0.03
	Reactome	✓	1288	53.3 (10 - 459)	0.63	6.93	0.83	-0.01
	WikiPathways	✓	621	47.4 (10 - 431)	0.45	4.11	0.88	-0.03
Gene Ontology	Biological process	✓	13915	69.7 (1 - 13687)	0.87	70.86	0.95	-0.00
	Molecular function	✓	4526	39.6 (1 - 14303)	0.91	12.55	0.95	-0.01
	Cellular component	✓	1834	150.5 (1 - 14238)	0.90	19.39	0.87	-0.01

Table S4: Descriptive statistics for the investigated module identification methods and pathway databases, filtered to modules with sizes ranging from 2 to 1000.

	Method	Overlaps	Count of identified modules	Average size (range)	Node coverage	Mean number of modules per gene	Conductance	Modularity overlap
General topology-based methods	CPM	✗	540	27.3 (2 - 830)	0.93	1.00	0.50	0.07
	Eigenvector	✗	2	883.5 (845 - 922)	0.11	1.00	0.29	0.01
	Greedy Modularity	✗	126	10.6 (2 - 244)	0.08	1.00	0.35	0.28
	Leiden	✗	25	225.5 (5 - 956)	0.36	1.00	0.17	0.19
	Louvain	✗	683	17.3 (2 - 834)	0.75	1.00	0.47	0.13
	Markov Clustering	✗	49	111.8 (2 - 868)	0.35	1.00	0.43	0.11
	Walktrap	✗	566	14.5 (2 - 826)	0.52	1.00	0.44	0.20
	ANGEL	✓	16	10.3 (4 - 47)	0.01	1.01	0.17	0.54
	ASLPAw	✓	369	22.4 (2 - 575)	0.50	1.05	0.49	0.13
	Core Expansion	✓	1974	20.5 (2 - 276)	0.82	3.12	0.81	0.06
	DPClus	✓	2486	6.0 (2 - 185)	0.77	1.22	0.78	-0.21
	Graph entropy	✓	3082	5.9 (2 - 198)	0.51	2.26	0.83	-0.24
	LFM	✓	289	47.7 (2 - 959)	0.65	1.35	0.44	0.08
	LPANNI	✓	895	20.7 (2 - 966)	0.89	1.32	0.56	0.14
	Node Perception	✓	8693	5.7 (2 - 978)	0.92	3.39	0.94	-0.10
	SLPA	✓	227	44.3 (2 - 722)	0.64	1.00	0.45	0.10
DREAM Challenge	DREAM K1	✗	581	27.2 (2 - 100)	1.00	1.00	0.59	0.08
	DREAM M1	✗	2029	7.8 (1 - 100)	1.00	1.00	0.85	-0.02
	DREAM R1	✗	327	44.7 (3 - 93)	0.93	1.00	0.61	0.04
UCoDe	Max. count: 500	✓	137	729.5 (5 - 998)	0.33	19.43	0.17	0.00
	Max. count: 600	✓	186	732.9 (12 - 993)	0.29	30.07	0.16	0.00
	Max. count: 700	✓	164	793.0 (5 - 1000)	0.18	45.78	0.14	0.00
	Max. count: 800	✓	130	719.8 (2 - 974)	0.19	31.13	0.11	-0.00
	Max. count: 900	✓	152	725.8 (9 - 993)	0.21	33.09	0.11	0.00
	Max. count: 1000	✓	209	715.3 (82 - 952)	0.23	40.97	0.14	0.00
NOCD	Max. count: 500	✓	5	699.2 (437 - 950)	0.21	1.06	0.37	0.03
	Max. count: 600	✓	3	318.3 (55 - 697)	0.06	1.00	0.28	0.04
	Max. count: 700	✓	6	769.2 (352 - 980)	0.26	1.11	0.34	0.03
	Max. count: 800	✓	5	717.0 (13 - 985)	0.21	1.06	0.41	0.03
	Max. count: 900	✓	4	671.8 (39 - 987)	0.16	1.07	0.28	0.04
	Max. count: 1000	✓	6	520.0 (7 - 877)	0.19	1.04	0.37	0.03
**GNN4DM**	Max. count: 500	✓	500	120.8 (6 - 659)	0.99	3.86	0.72	-0.00
	Max. count: 600	✓	600	102.3 (4 - 603)	0.99	3.91	0.73	-0.00
	Max. count: 700	✓	700	95.4 (4 - 527)	0.99	4.26	0.73	-0.00
	Max. count: 800	✓	800	81.8 (4 - 666)	0.99	4.17	0.75	-0.01
	Max. count: 900	✓	900	75.0 (2 - 462)	0.99	4.30	0.76	-0.01
	Max. count: 1000	✓	993	72.3 (3 - 544)	0.99	4.57	0.76	-0.01
Pathways	BioCarta	✓	221	19.0 (10 - 78)	0.08	3.17	0.94	-0.10
	KEGG	✓	186	66.0 (10 - 321)	0.31	2.51	0.77	-0.03
	Reactome	✓	1288	53.3 (10 - 459)	0.63	6.93	0.83	-0.01
	WikiPathways	✓	621	47.4 (10 - 431)	0.45	4.11	0.88	-0.03
Gene Ontology	Biological process	✓	10995	42.2 (2 - 995)	0.83	35.62	0.94	-0.00
	Molecular function	✓	3081	32.1 (2 - 991)	0.74	8.47	0.93	-0.03
	Cellular component	✓	1557	48.1 (2 - 945)	0.64	7.44	0.87	-0.06

Table S5: Final setting of hyper-parameters in GNN4DM.

Hyper-parameter	Value
Number of GCN layers	2
Size of hidden GCN layer	128
Activation functions	(1) ReLU, (2) SoftPlus
BCE loss weight (λ)	10.0
Optimizer	AdamW
Learning rate	0.001
Learning rate scheduler	Step (γ: 0.85, step size: 300)

4 Supplementary Figures

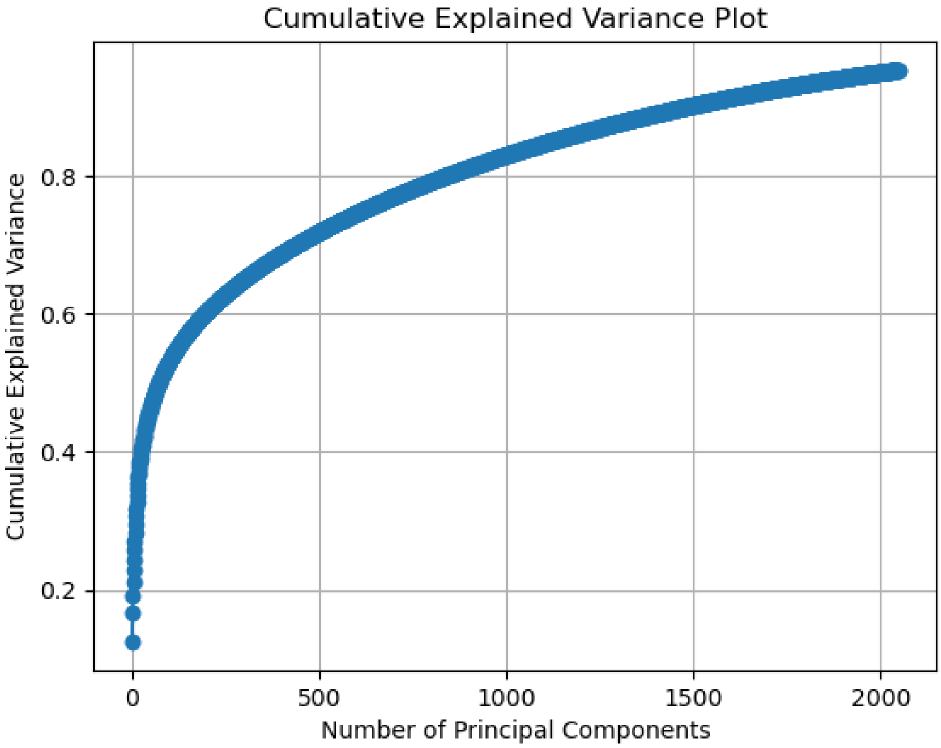

Figure S1: Cumulative explained variance of the non-UK Biobank GWAS summary statistics as a function of the number of principal components (PCs) used. Gene-level genome-wide association data was originated from the GWAS Atlas project Release 3, which encompasses 4756 publicly available GWAS summary statistics. We downloaded the dataset containing the gene-level p-values computed by the MAGMA software for 19 436 protein-coding genes. We split the dataset into two parts; 3545 summary statistics exclusive to studies other than UK Biobank were employed as inputs, while the 1211 UK Biobank-specific summary statistics were utilized for evaluation purposes. We imputed the missing p-values for 519 genes absent in the dataset with 0.5. We computed the negative log-transformed p-values and performed a PCA transformation for dimensionality- and noise reduction of the resulting significance values. The plot shows that at 512 PCs, the cumulative explained variance is 71.8%, while at 1024 PCs, it increases to 83.1%, and at 2048 PCs, it reaches 95.3%. We selected 512 PCs as a balance between capturing a relatively high amount of variance and maintaining a manageable number of input features. Increasing the number of PCs beyond 512 would result in diminishing returns in explained variance, with only an additional 11.3% gain when using 1024 PCs, at the cost of significantly increasing the model size, which could lead to overfitting issues.

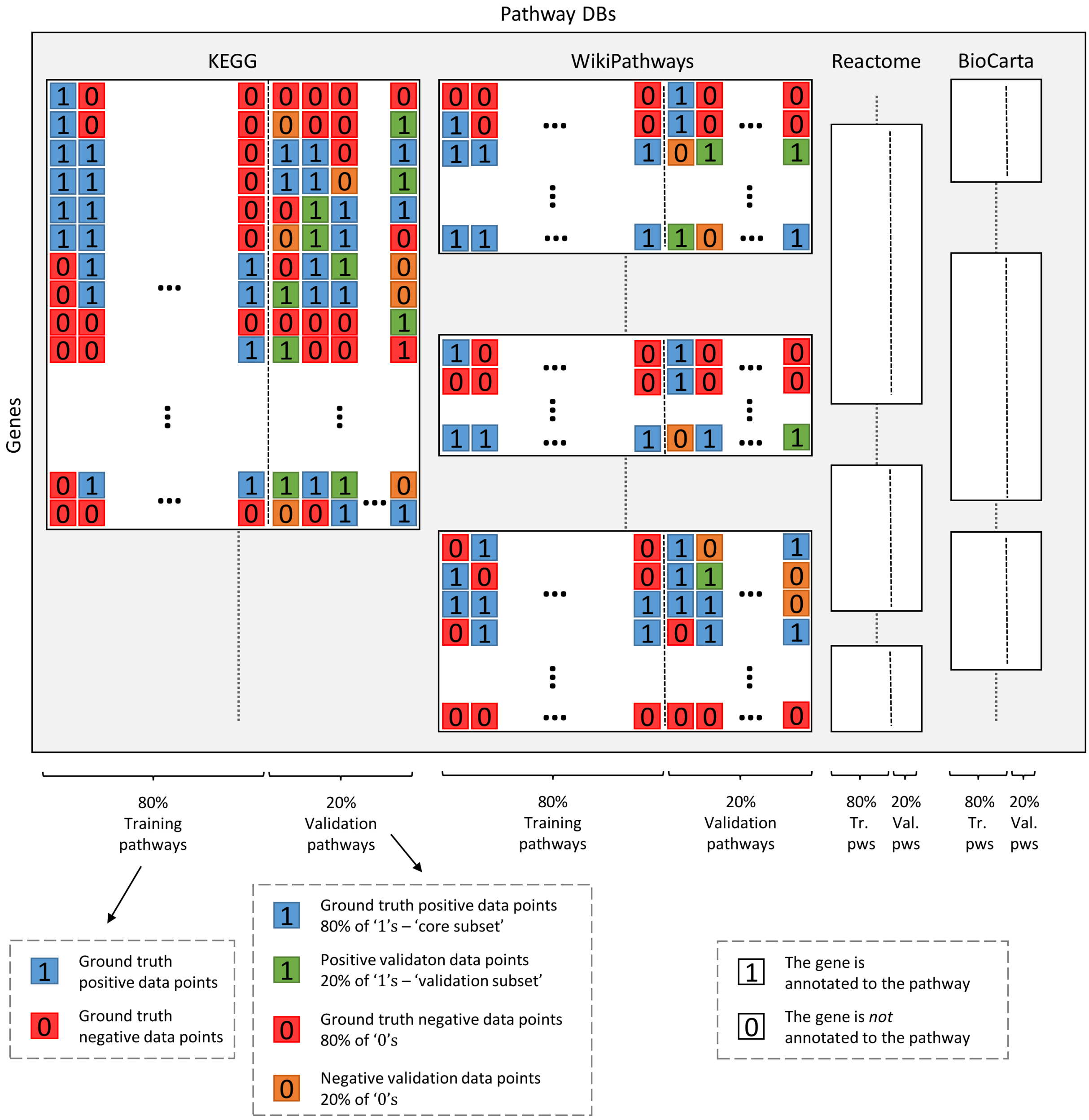

Figure S2: Illustration of the train-validation split used in the model. In the data matrix, rows correspond to genes, and columns correspond to pathways, grouped by the pathway database (indicated by white boxes). Before training, pathways in each database are split into two disjoint sets: 80% as 'training pathways' used intact during training, and 20% as 'validation pathways' utilized separately for training and validation. '1's represent gene-pathway annotations, while '0's indicate missing annotations (i.e., a gene is not annotated to a pathway). For training pathways, all annotated genes are ground truth positive data points, and other genes with at least one annotation in the database are ground truth negative data points. In validation pathways, annotated genes are split into a core subset (80%) for training and a validation subset (20%) left out during training. Non-annotated genes are similarly split into training and validation sets. The core subset guides the model in predicting specific pathways during training. Training loss is calculated on ground truth data points (indicated with blue and red) from both training and validation pathways, while validation loss is measured on validation data points (indicated with green and orange). The mosaic pattern in rows illustrates the overlap of genes across different pathway databases. KEGG and WikiPathways are illustrated with detailed examples, while Reactome and BioCarta are shown schematically.

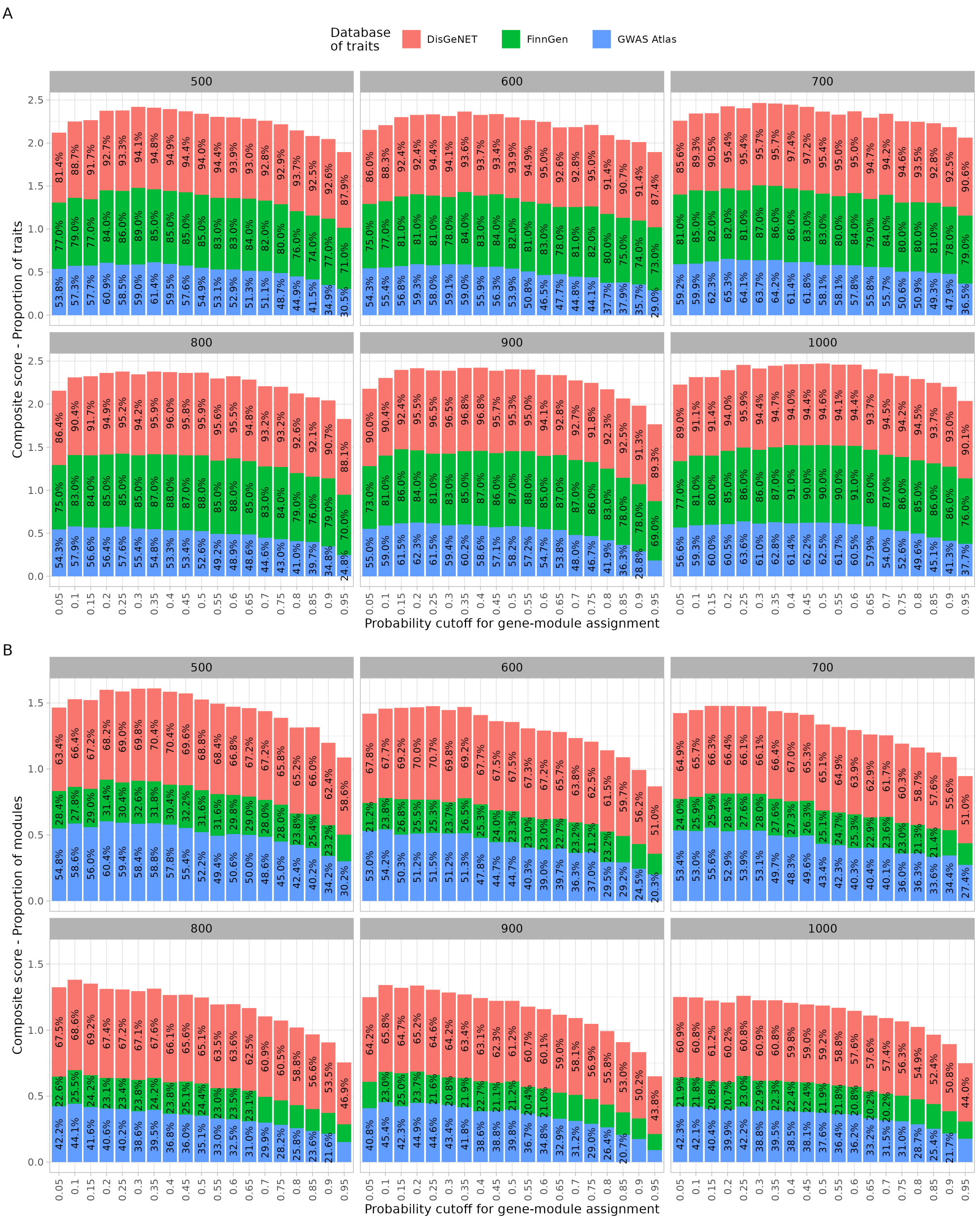

Figure S3: The effect of varying probability cutoffs on gene-module assignment and enrichment scores across different databases and module counts. (**A**) Composite scores defined by the cumulative proportion of traits for which at least one significant module was identified. (**B**) Composite scores defined by the cumulative proportion of modules for which at least one significant trait was identified. The evaluation was performed at different probability cutoff values, ranging from 0.05 to 0.95 in increments of 0.05. Column groups indicate module count setting. Column colors indicate the evaluation datasets: DisGeNET (red), FinnGen (green), and GWAS Atlas (blue). Percentage values inside the columns show the proportion of traits (in A), or modules (in B) that are significantly enriched/over-represented in the corresponding trait data source. Only percentages greater than 20% are shown.

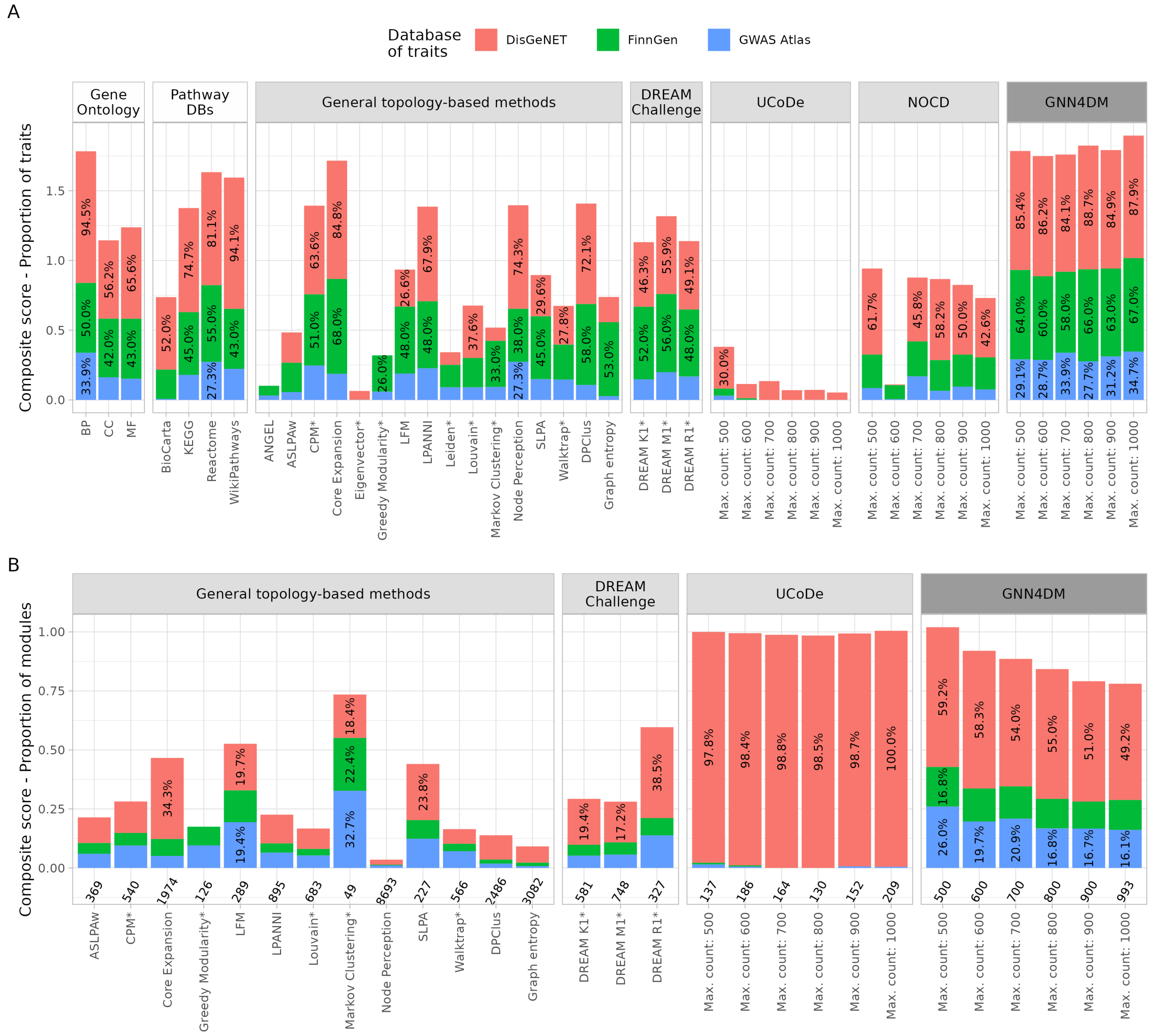

Figure S4: Performance evaluation of module identification methods and pathway databases using significance threshold FDR = 0.01. See the description of Figure 2 for details.

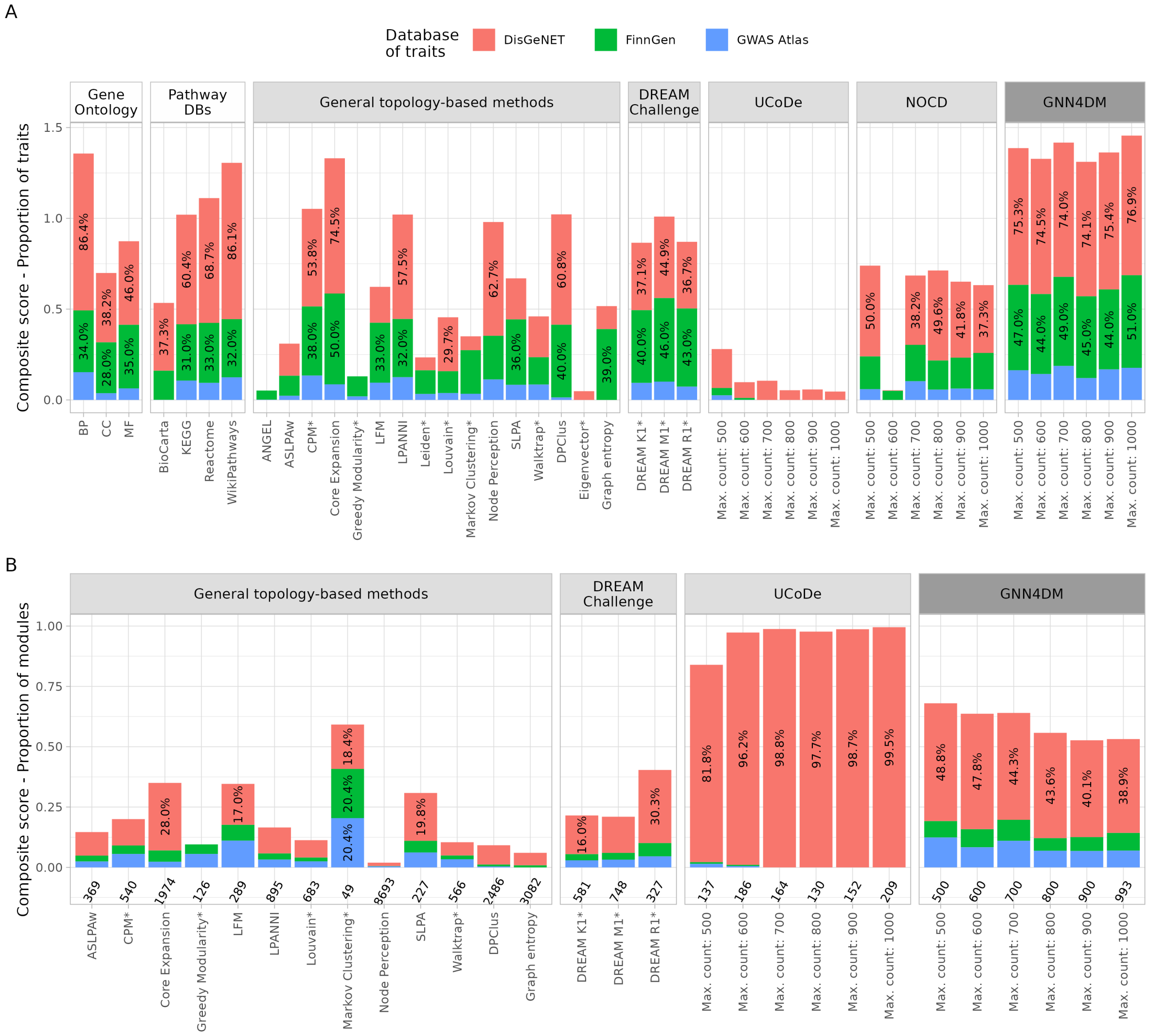

Figure S5: Performance evaluation of module identification methods and pathway databases using significance threshold FDR = 0.001. See the description of Figure 2 for details.

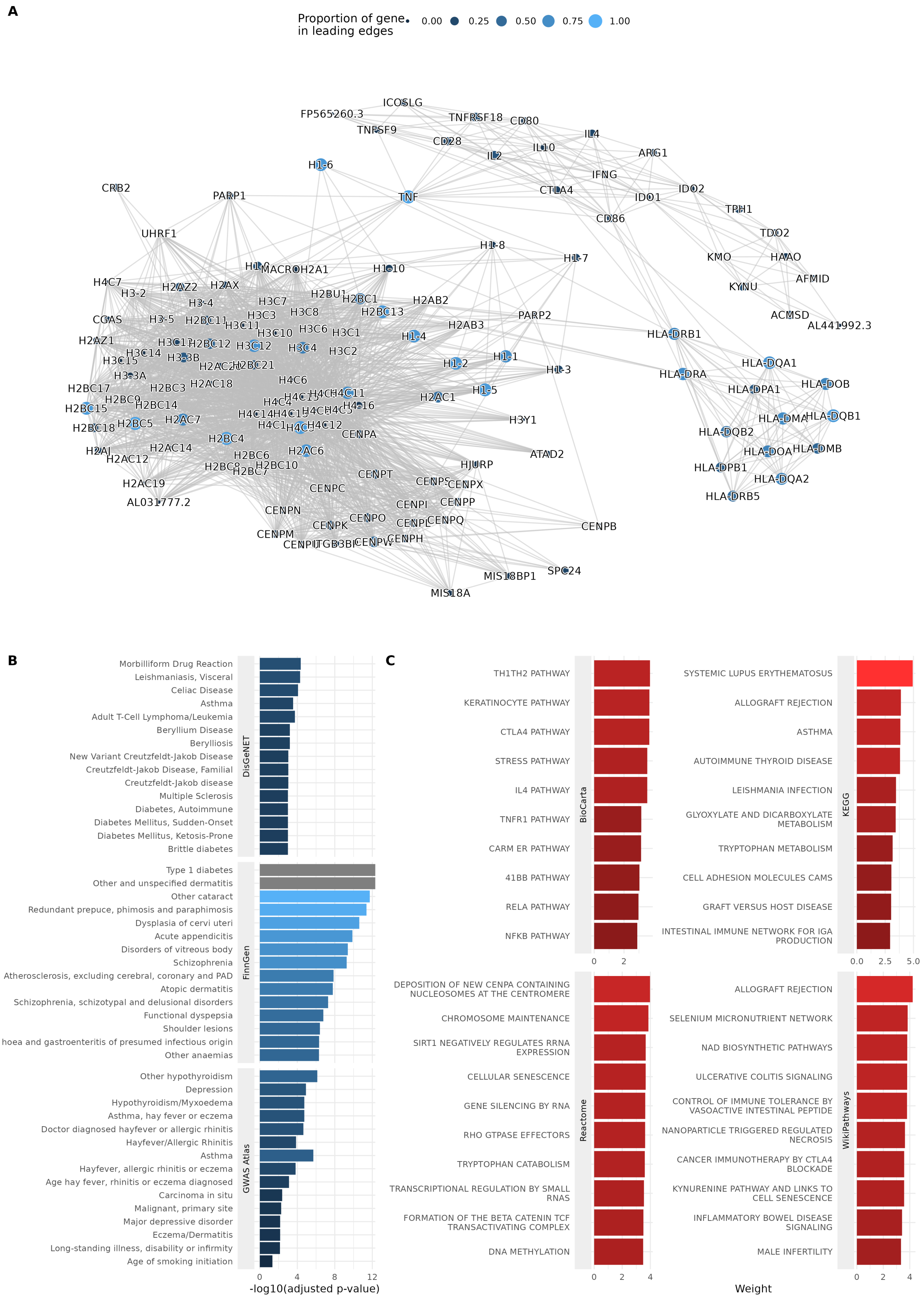

Figure S6: A multimorbidity module significantly enriched in multiple diseases. (**A**) The graph displays a subnetwork of the STRING PPI network, representing the module. The size and color of each node denote the gene's proportion among the leading edge genes for those diseases in which the module is significantly enriched, as determined by GWAS Atlas. (**B**) The top 15 diseases significantly associated with the module, as identified in the three evaluation datasets. (**C**) The top 10 pathways in each pathway database that are relevant to the module. The bars show the weight GNN4DM learned for the module in association with the corresponding pathway.

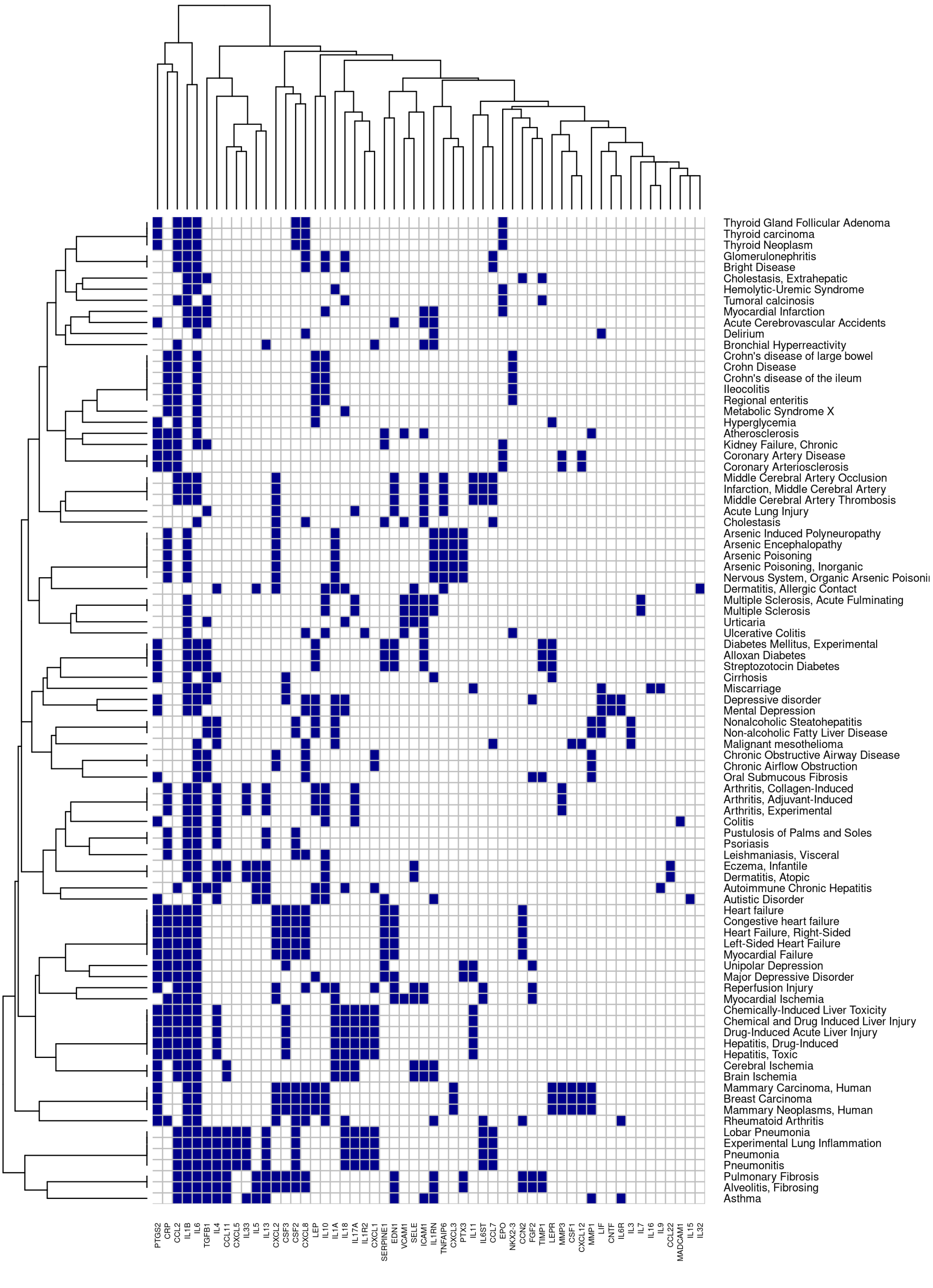

Figure S7: Overlap between genes of the module shown in Figure 3 and the genes related to the disorders significantly associated with the module. Gene-disease associations originate from the DisGeNET database. Only disorders with FDR$<$ 0.0001 and their related genes are shown. Both genes and disorders are hierarchically clustered using Ward's method.

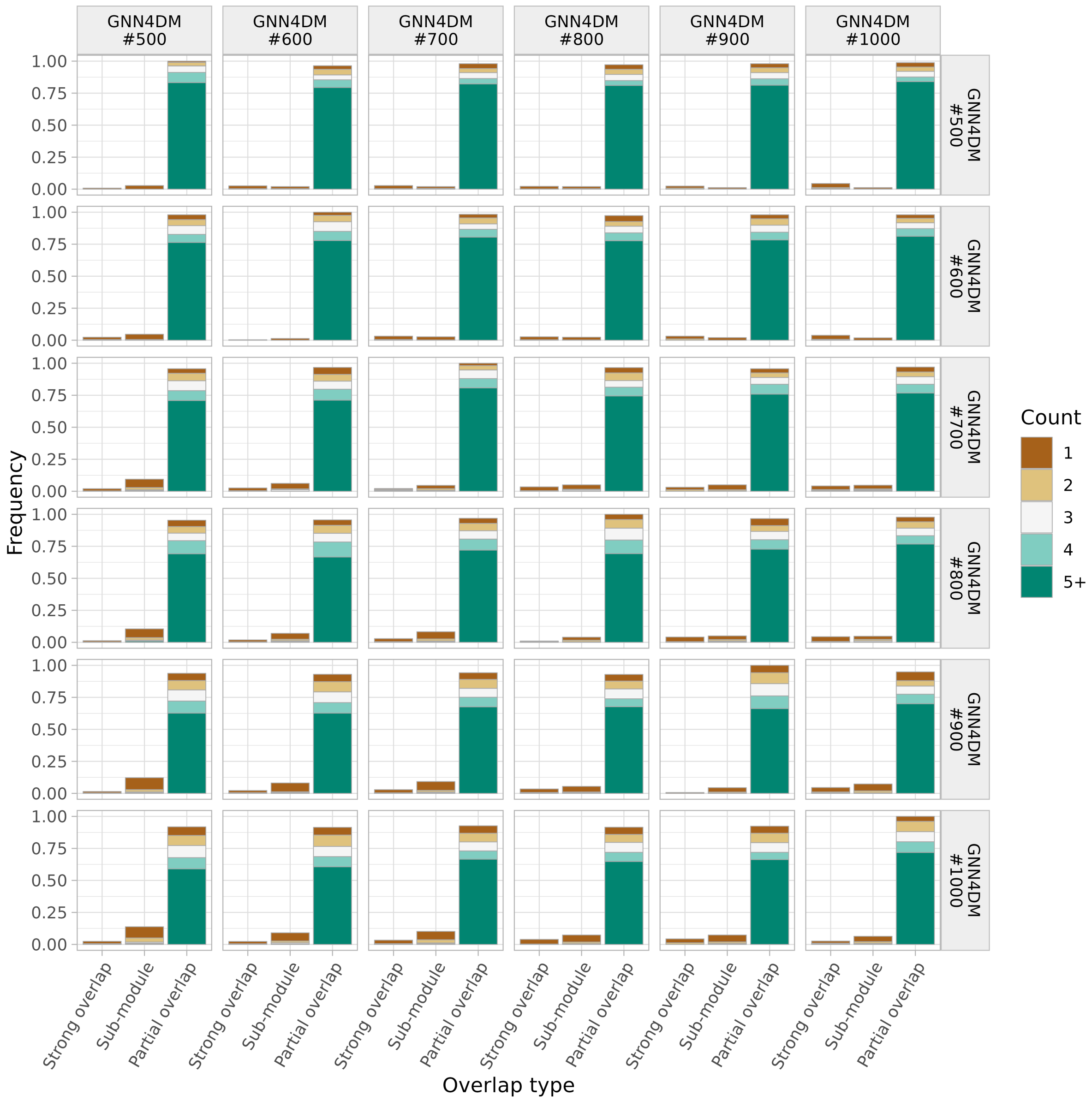

Figure S8: Characterization of all pairwise overlaps between GNN4DM-identified modules. For each row, we consider all modules identified using the corresponding module count setting and characterize their overlaps with those indicated in each column. See Supplementary Methods for the definition of the various overlap types.

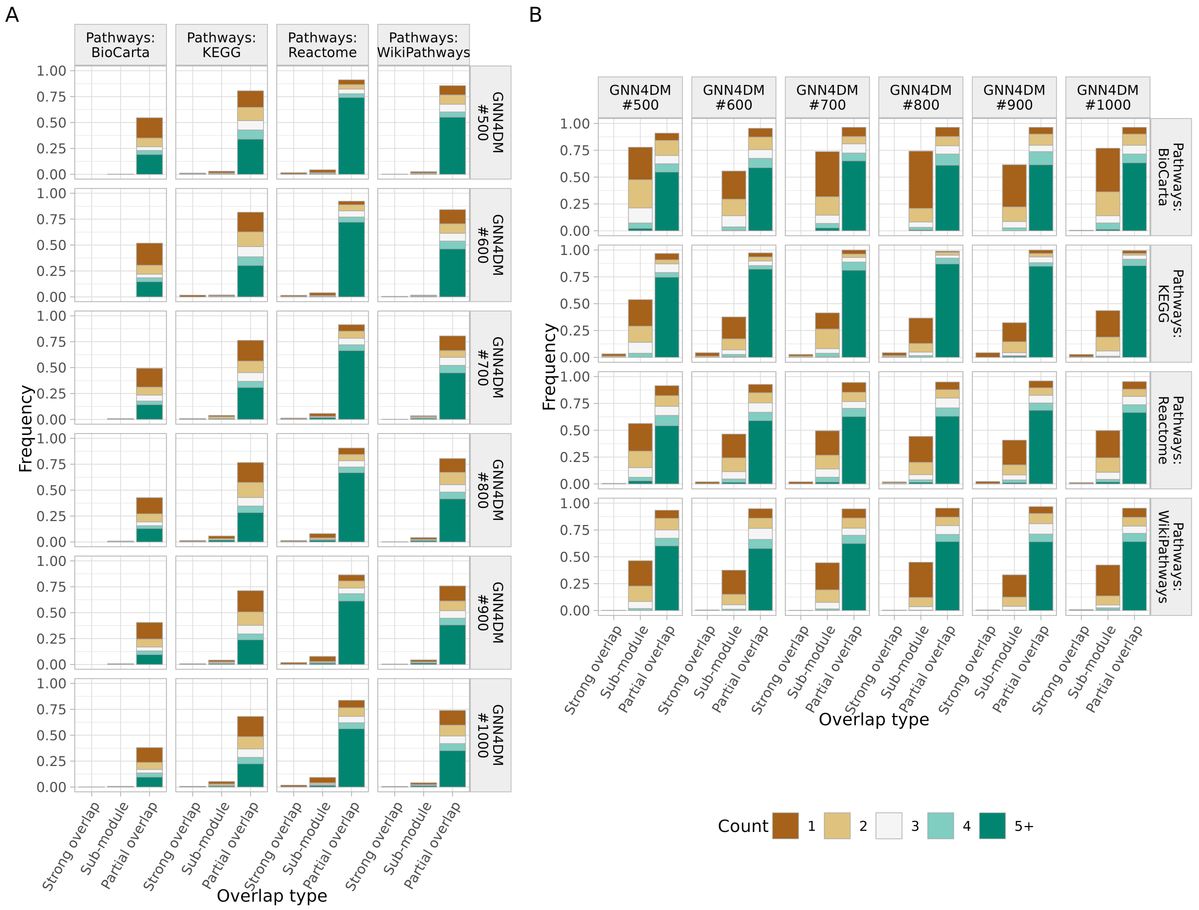

Figure S9: Characterization of all pairwise overlaps between GNN4DM-identified modules and pathway databases. See Supplementary Methods for the definition of the various overlap types. **(A)** For each row, we consider all modules identified by GNN4DM by the corresponding module count setting and characterize their overlaps with the pathways from the database in each column. **(B)** For each row, we consider all pathways from the corresponding database and characterize their overlaps with the modules identified by GNN4DM by the corresponding module count setting in each column.

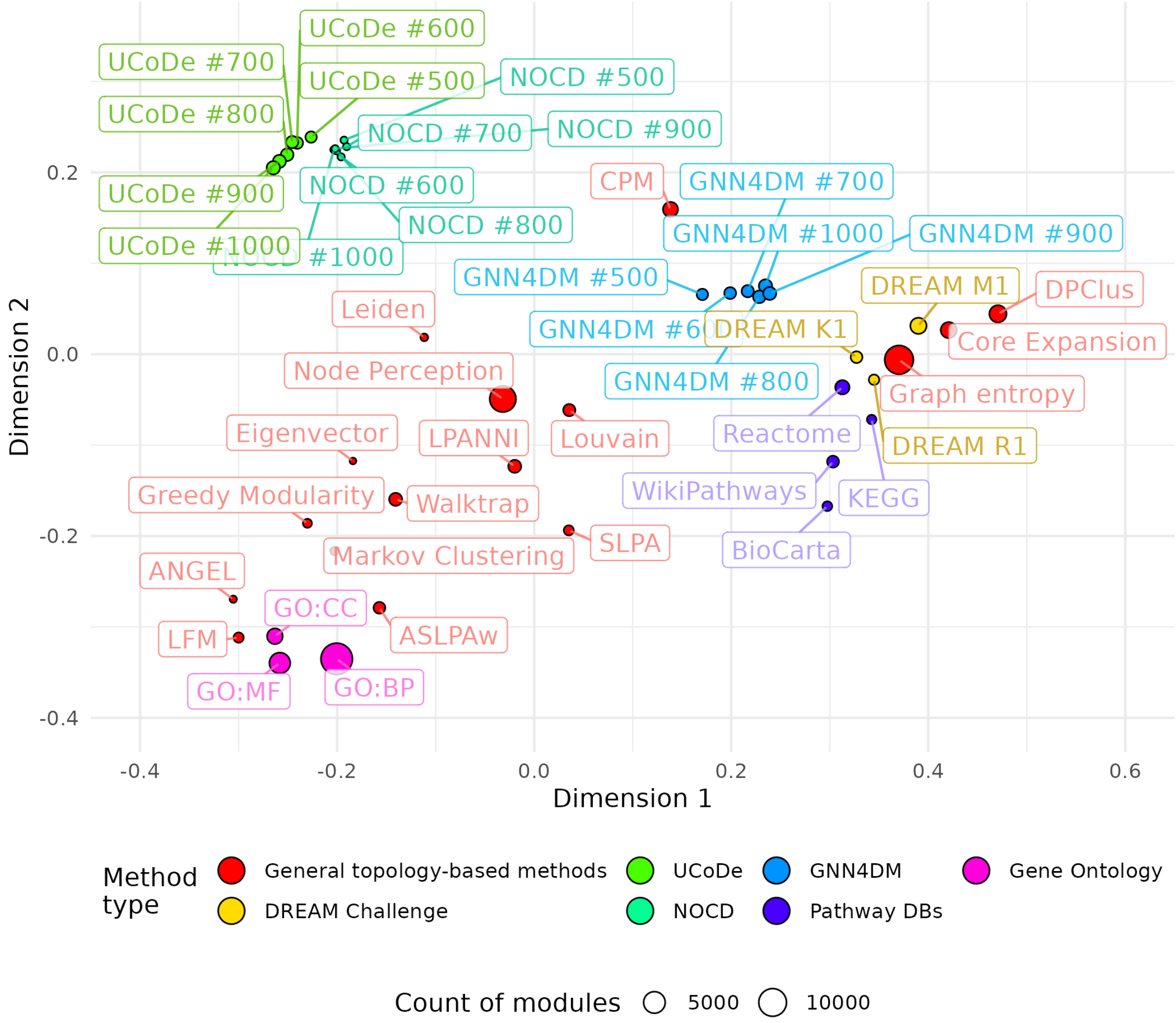

Figure S10: Similarity and complementarity of different module identification methods. Each point corresponds to a module identification method or pathway database. The proximity of two points in the plot indicates a higher similarity in their corresponding module predictions (multidimensional scaling, see Supplementary Methods). The size of the points corresponds to the count of identified modules. The points' and labels' color indicates the type of the methods.

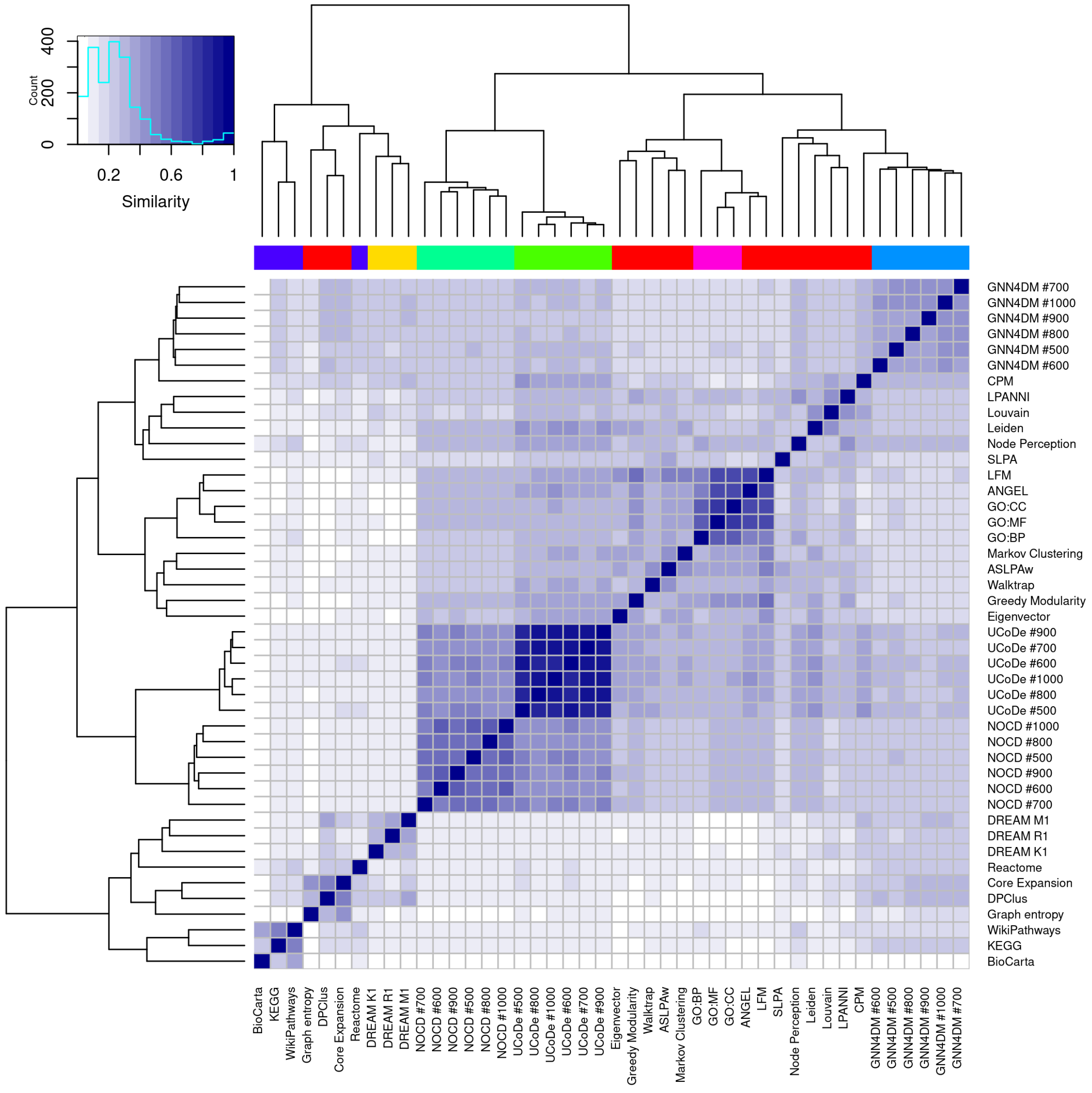

Figure S11: Pairwise similarity of different module identification methods. Similarity was computed based on whether the same genes were clustered together by the two methods. Specifically, a prediction vector was defined for every method, specifying for every gene pair how many modules they were co-clustered in (see Supplementary Methods). A corresponding distance matrix between all methods was computed as the inverse cosine similarity of the prediction vectors and hierarchically clustered using Ward's method. The annotation row indicates the method type.

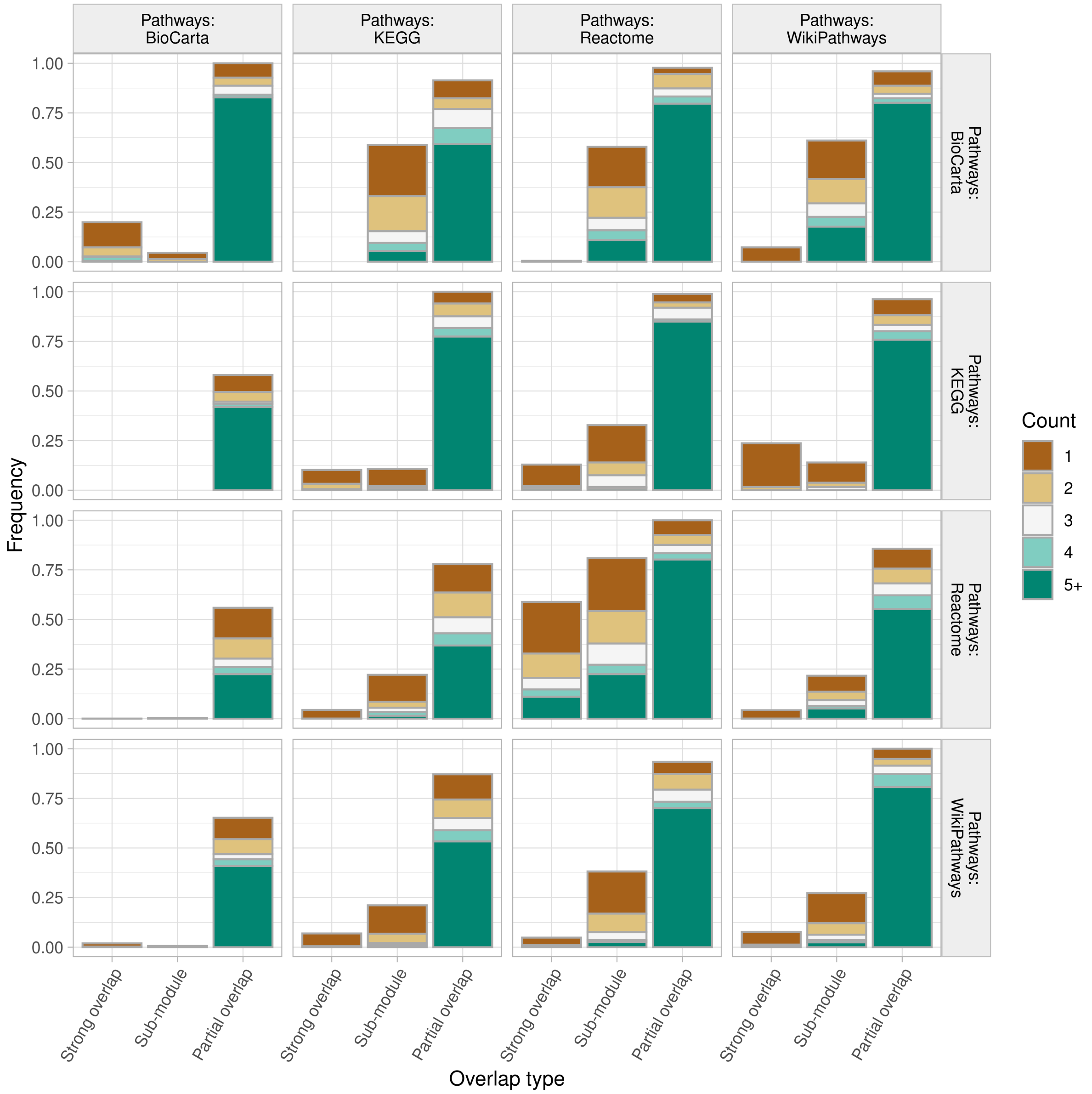

Figure S12: Characterization of all pairwise overlaps between pathways in various pathway databases used for training the model. For each row, we consider all pathways from the corresponding database and characterize their overlaps with those from the database in each column. See Supplementary Methods for the definition of the various overlap types.

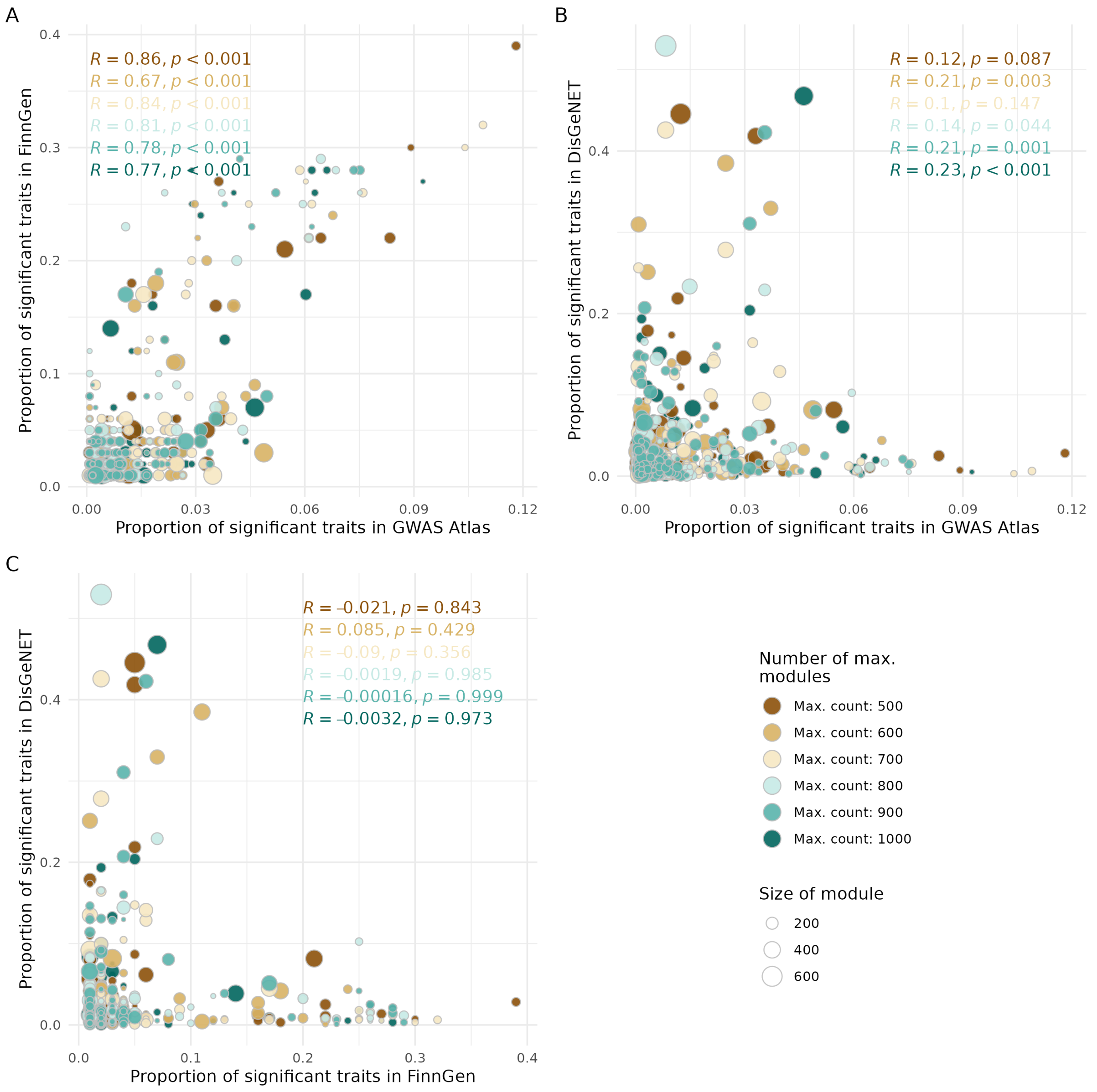

Figure S13: Scatter plots of identified modules according to GNN4DM. Each point corresponds to a module identified by GNN4DM. The color of the nodes denote the maximum module count parameter of the model. The size of the nodes indicates the number of genes assigned to the module. The Pearson correlation coefficient and its associated p-values are shown, indicating the extent of correlation across the evaluation datasets.

References

[1] Md Altaf-Ul-Amin, Yoko Shinbo, Kenji Mihara, Ken Kurokawa, and Shigehiko Kanaya. Development and implementation of an algorithm for detection of protein complexes in large interaction networks. *BMC Bioinformatics*, 7:207, 12 2006.

[2] Vincent D Blondel, Jean-Loup Guillaume, Renaud Lambiotte, and Etienne Lefebvre. Fast unfolding of communities in large networks. *Journal of Statistical Mechanics: Theory and Experiment*, 2008:P10008, 10 2008.

[3] Sarvenaz Choobdar, Mehmet E. Ahsen, Jake Crawford, Mattia Tomasoni, Tao Fang, David Lamparter, Junyuan Lin, Benjamin Hescott, Xiaozhe Hu, Johnathan Mercer, Ted Natoli, Rajiv Narayan, Aravind Subramanian, Jitao D. Zhang, Gustavo Stolovitzky, Zoltán Kutalik, Kasper Lage, Donna K. Slonim, Julio Saez-Rodriguez, Lenore J. Cowen, Sven Bergmann, and Daniel Marbach. Assessment of network module identification across complex diseases. *Nature Methods*, 16:843–852, 9 2019.

[4] Ali Choumane, Ali Awada, and Ali Harkous. Core expansion: a new community detection algorithm based on neighborhood overlap. *Social Network Analysis and Mining*, 10:30, 12 2020.

[5] Aaron Clauset, M. E. J. Newman, and Cristopher Moore. Finding community structure in very large networks. *Physical Review E*, 70:066111, 12 2004.

[6] A J Enright, S Van Dongen, and C A Ouzounis. An efficient algorithm for large-scale detection of protein families. *Nucleic acids research*, 30:1575–84, 4 2002.

[7] Edward Casey Kenley and Young-Rae Cho. Detecting protein complexes and functional modules from protein interaction networks: A graph entropy approach. *PROTEOMICS*, 11:3835–3844, 10 2011.

[8] Andrea Lancichinetti, Santo Fortunato, and János Kertész. Detecting the overlapping and hierarchical community structure in complex networks. *New Journal of Physics*, 11:033015, 3 2009.

[9] Meilian Lu, Zhenglin Zhang, Zhihe Qu, and Yu Kang. Lpanni: Overlapping community detection using label propagation in large-scale complex networks. *IEEE Transactions on Knowledge and Data Engineering*, 31:1736–1749, 9 2019.

[10] M. E. J. Newman. Finding community structure in networks using the eigenvectors of matrices. *Physical Review E*, 74:036104, 9 2006.

[11] Pascal Pons and Matthieu Latapy. Computing communities in large networks using random walks. In pInar Yolum, Tunga Güngör, Fikret Gürgen, and Can Özturan,

editors, *Computer and Information Sciences - ISCIS 2005*, pages 284–293, Berlin, Heidelberg, 2005. Springer Berlin Heidelberg.

[12] Giulio Rossetti. Angel: efficient, and effective, node-centric community discovery in static and dynamic networks. *Applied Network Science*, 5:26, 12 2020.

[13] Sucheta Soundarajan and John E. Hopcroft. Use of local group information to identify communities in networks. *ACM Transactions on Knowledge Discovery from Data*, 9:1–27, 4 2015.

[14] Mattia Tomasoni, Sergio Gómez, Jake Crawford, Weijia Zhang, Sarvenaz Choobdar, Daniel Marbach, and Sven Bergmann. Monet: a toolbox integrating top-performing methods for network modularization. *Bioinformatics*, 36:3920–3921, 6 2020.

[15] V. A. Traag, P. Van Dooren, and Y. Nesterov. Narrow scope for resolution-limit-free community detection. *Physical Review E*, 84:016114, 7 2011.

[16] Vincent Antonio Traag, Ludo Waltman, and Nees Jan van Eck. From louvain to leiden: guaranteeing well-connected communities. *Scientific Reports*, 9, 2018.

[17] Jierui Xie, Boleslaw K. Szymanski, and Xiaoming Liu. Slpa: Uncovering overlapping communities in social networks via a speaker-listener interaction dynamic process. In *2011 IEEE 11th International Conference on Data Mining Workshops*, pages 344–349, 2011.
